# The diagnostic potential of multimodal neuroimaging measures in Parkinson's disease and atypical parkinsonism

**DOI:** 10.1002/brb3.1808

**Published:** 2020-10-07

**Authors:** Chang‐hyun Park, Phil Hyu Lee, Seung‐Koo Lee, Seok Jong Chung, Na‐Young Shin

**Affiliations:** ^1^ Department of Radiology College of Medicine, The Catholic University of Korea Seoul Korea; ^2^ Center for Neuroprosthetics and Brain Mind Institute Swiss Federal Institute of Technology (EPFL) Geneva Switzerland; ^3^ Department of Neurology Yonsei University College of Medicine Seoul Korea; ^4^ Department of Radiology Yonsei University College of Medicine Seoul Korea; ^5^ Department of Neurology Yongin Severance Hospital Yonsei University Health System Yongin Korea

**Keywords:** functional MRI, machine learning, multiple system atrophy, Parkinson's disease, progressive supranuclear palsy, structural MRI

## Abstract

**Introduction:**

For the diagnosis of Parkinson's disease (PD) and atypical parkinsonism (AP) using neuroimaging, structural measures have been largely employed since structural abnormalities are most noticeable in the diseases. Functional abnormalities have been known as well, though less clearly seen, and thus, the addition of functional measures to structural measures is expected to be more informative for the diagnosis. Here, we aimed to assess whether multimodal neuroimaging measures of structural and functional alterations could have potential for enhancing performance in diverse diagnostic classification problems.

**Methods:**

For 77 patients with PD, 86 patients with AP comprising multiple system atrophy and progressive supranuclear palsy, and 53 healthy controls (HC), structural and functional MRI data were collected. Gray matter (GM) volume was acquired as a structural measure, and GM regional homogeneity and degree centrality were acquired as functional measures. The measures were used as predictors individually or in combination in support vector machine classifiers for different problems of distinguishing between HC and each diagnostic type and between different diagnostic types.

**Results:**

In statistical comparisons of the measures, structural alterations were extensively seen in all diagnostic types, whereas functional alterations were limited to specific diagnostic types. The addition of functional measures to the structural measure generally yielded statistically significant improvements to classification accuracy, compared to the use of the structural measure alone.

**Conclusion:**

We suggest the fusion of multimodal neuroimaging measures as an effective strategy that could generally cope with diverse prediction problems of clinical concerns.

## Graphical abstract

The addition of functional measures (ReHo and DegCen) to the structural measure (Vol) generally yielded statistically significant improvements to classification accuracy, compared to the use of the structural measure alone, across different problems of distinguishing between healthy controls (HC) and each diagnostic type of parkinsonism (PD, MSA, and PSP) and between different diagnostic types of parkinsonism.

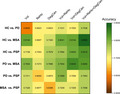

## 1 INTRODUCTION

In the era of precision medicine, machine learning‐based predictive modeling has great potential for clinical prediction problems including the diagnosis of specific diseases, as it can find patterns that a single clinician may not detect when it is applied to various clinical data sources. However, there are also challenges regarding a lack of its meaningful contributions to clinical practice primarily due to the need of additional measures characterizing the disease process as well as the demand for a large sample size (Deo, 2015).

For the diagnosis of Parkinson's disease (PD) and atypical parkinsonism (AP), though still usually reliant on a medical history and neurological examination in clinical practice, neuroimaging measures have been increasingly employed. Furthermore, the application of machine learning to neuroimaging measures has shown improved performance in classification problems related to the diagnosis (Rana et al., 2015; Salvatore et al., 2014). In particular, given challenges of the differential diagnosis between PD and AP as often manifested as the underdiagnosis of AP and the overdiagnosis of PD (Irene Litvan, 1999), machine learning‐based predictive modeling with neuroimaging measures has emerged as an approach to discover new insights into the diagnosis (Garraux et al., 2013; Scherfler et al., 2016).

Among other MRI measures, gray matter (GM) volume acquired from structural MRI (sMRI) has been successfully employed for machine learning by taking account of structural abnormalities, specifically GM atrophy, in PD (Brenneis et al., 2003; Summerfield et al., 2005) and AP (Brenneis et al., 2003; Messina et al., 2011). In contrast, despite possible functional abnormalities related to PD and AP (Choe, Yeo, Chung, Kim, & Lim, 2013; Fang et al., 2017; Li, Liang, Jia, & Li, 2016; Wu et al., 2009), the potential of functional measures acquired from functional MRI (fMRI) for the diagnosis of the diseases at a single individual level has been rarely examined. Moreover, it remains unclear whether the fusion of structural and functional measures could be more informative than the use of structural measures alone especially when diverse diagnostic classification problems are considered.

In this study, we proposed three kinds of diagnostic classification problems: (a) discriminating PD and AP from healthy controls (HC); (b) distinguishing between PD and AP; and (c) classifying subtypes of AP. We wanted to search for an effective strategy that would generally cope with such diverse diagnostic classification problems. Specifically, we sought to test whether multimodal neuroimaging measures of structural and functional abnormalities could help to enhance classification performance compared to the use of monomodal neuroimaging measures alone.

## 2 METHODS

### 2.1 Participants

From the movement disorders and dementia database collected prospectively from 2011 to 2016 at a single tertiary hospital, patients who underwent neurological examination, including the unified Parkinson's disease rating scale (UPDRS) (Fahn, Elton, & Members of the UPDRS Development Committee, 1987) and mini‐mental state examination (MMSE), and conventional MRI scans at the first visit to the clinic, were selected. At least 3 years after the first visit, the most up‐to‐date diagnosis was retrieved from the case files of each patient. To ensure a differential diagnosis, apart from consensus criteria (Bensimon et al., 2008; Gelb, Oliver, & Gilman, 1999; Hughes, Daniel, Kilford, & Lees, 1992; Litvan et al., 1996), additional imaging modalities, such as ^18^F‐*N*‐(3‐fluoropropyl)‐2β‐carboxymethoxy‐3β‐(4‐iodophenyl) nortropane PET, ^18^F‐fluorodeoxyglucose PET, and cardiac metaiodobenzylguanidine imaging, were adopted if needed and clinical features and drug responses during a follow‐up were considered.

As diagnostic results, 77 patients (67.62 ± 7.72 years, 33 females and 44 males) were diagnosed with PD and 86 patients (66.28 ± 9.24 years, 37 females and 49 males) were diagnosed with probable AP. The patients who were diagnosed with AP were further divided into 44 patients (61.73 ± 9.19 years, 18 females and 26 males) with multiple system atrophy (MSA) and 42 patients (71.04 ± 6.56 years, 19 females and 23 males) with progressive supranuclear palsy (PSP). The patients with MSA included both phenotypes: the parkinsonian variant (MSA‐P, 21 patients) and cerebellar variant (MSA‐C, 21 patients) (Gilman et al., 1999). Fifty‐three age‐ and sex‐matched healthy participants (66.87 ± 8.36 years, 28 females and 25 males) were also recruited, and they served as HC. This retrospective study was approved by the Yonsei University Health System institutional review board, and a waiver of informed consent was obtained.

### 2.2 Acquisition and processing of neuroimaging data

MRI scans were collected using an Achieva 3 T MRI system (Philips Healthcare). Structural MRI (sMRI) data of one volume were acquired in coronal planes with a 3D T1‐weighted SENSE parallel imaging sequence: number of slices = 210, slice thickness = 1.00 mm, matrix size = 256 × 256, and in‐plane resolution = 0.875 mm × 0.875 mm. Resting state fMRI (rsfMRI) data of 165 volumes were obtained in axial planes with a T2*‐weighted gradient‐echo echo‐planar imaging sequence: repetition time = 2,000 ms, echo time = 30 ms, number of slices = 31, slice thickness = 4.00 mm, matrix size = 80 × 80, and in‐plane resolution = 2.75 mm × 2.75 mm. Using the tools in SPM12 (http://www.fil.ion.ucl.ac.uk/spm/, RRID:SCR_007037) and DPARSF (http://rfmri.org/DPARSF/, RRID:SCR_002372), sMRI and rsfMRI data were preprocessed. GM volume, as a main measure indicating structural abnormalities in PD and AP, was acquired from sMRI. In addition, as measures representing functional abnormalities at local and global levels, regional homogeneity (ReHo) (Zang, Jiang, Lu, He, & Tian, 2004) and degree centrality (DegCen), respectively, were obtained from rsfMRI. Details on how the neuroimaging data were processed to acquire the structural and functional measures are described in Appendix S1.

### 2.3 Statistical inferences on neuroimaging measures

At a group level, differences in the voxel‐wise measures, including GM volume, ReHo, and DegCen, were inferred using two‐sample *t* tests between HC and each diagnostic type and between different diagnostic types. In the voxel‐wise statistical inferences, influences of age, sex, and years of education were adjusted commonly for the three measures, and an effect of total intracranial volume (TIV) was additionally adjusted for GM volume. Statistical significance was determined at an extent threshold of a *p* value of .05 family‐wise error corrected for multiple comparisons with a height threshold of a *p* value of .001.

### 2.4 Generation of predictor sets and application of machine learning

The parcellation of 120 GM regions was determined according to the modified automated anatomical labeling (AAL) atlas (Rolls, Joliot, & Tzourio‐Mazoyer, 2015). They contain 94 cerebral regions and 26 cerebellar regions (Table S1). For each GM region, GM volume divided by TIV, ReHo, and DegCen averaged over voxels within the region were assigned. Since the collection of values according to the choice of a specific atlas seems to be arbitrary in acquiring predictor values, we also applied the same predictive modeling procedure described below to the choice of different atlases. Details on the parcellation of GM regions according to different atlases are described in Appendix S1.

For tasks of classification between HC and each diagnostic type and between different diagnostic types, we employed the support vector machine (SVM) as a machine learning method. Having trained an SVM classifier for each classification problem, we evaluated classification accuracy via leave‐one‐out cross‐validation, by which classification accuracy was computed for each left‐out instance other than instances used for training. To assess improvements to classification accuracy according to the combined use of multimodal neuroimaging measures in developing SVM classifiers, we considered combinations of more than two measures as well as individual measures as predictor sets. Specifically, the fusion of the structural and functional measures was manifested by combining GM volume and ReHo, by combining GM volume and DegCen, and by combining all the three measures. Each of the predictor sets was corrected for effects of age, sex, and years of education by obtaining residuals after regressing out the confounding covariates. To reduce the risk of overfitting, irrelevant predictors were removed when they failed to pass a criterion of showing a difference between two groups with a *p* value of.05 uncorrected for multiple comparisons in a two‐sample *t* test.

### 2.5 Comparison of classification accuracy

To compare classification accuracy between different SVM classifiers, specifically between the one constructed with the structural measure alone and the ones constructed by the combination of the structural and functional measures, we used a resampling approach (Hothorn, Leisch, Zeileis, & Hornik, 2005) to derive a distribution of classification accuracy. Resampling was performed by applying 10‐fold cross‐validations iteratively 1,000 times, such that 10,000 estimates of classification accuracy were collected. The distribution of the estimates was represented as a curve the shape of which has been defined by a kernel smoothing function. With matched resampling for two SVM classifiers, one‐sided one‐sample *t* tests were conducted to assess the null hypothesis of zero or negative differences in the estimates. Statistical significance was determined at a *p* value of .05 family‐wise error corrected for multiple comparisons.

### 2.6 Identification of GM regions contributing to classification

For the SVM classifier that has been composed by the combination of all the three measures, we identified GM regions that contributed to classification and assessed how repeatedly each GM region was involved in different measures. In addition, as crucial roles of the cerebellum in PD and AP have been noted (Wu & Hallett, 2013), we computed relative weight ratios of cerebral and cerebellar regions by segregating GM regions involved in each measure into the two regions.

## 3 RESULTS

### 3.1 Demographic and clinical characteristics

Table 1 summarizes demographic and clinical characteristics of all participants. The patients were matched in age and sex with HC despite PSP's being relatively older among the patients. Motor function, as assessed with the motor section of the UPDRS, was not different between different diagnostic types. Cognitive function, as assessed with the MMSE, was higher than 24 out of 30 points in all diagnostic types, indicating the patients' cognition not being severely abnormal, although a statistical difference was seen between PD and AP.

**TABLE 1:** Demographic and clinical characteristics of participants

	HC	Patients			*p* value		
		PD	AP		HC versus. Patients	PD versus. AP	MSA versus. PSP
			MSA	PSP			
Sample size	53	77	44	42			
Age, years (mean ± *SD*)	66.87 ± 8.36	67.62 ± 7.72	61.73 ± 9.19	71.04 ± 6.56	NS	NS	<.001
Sex (female:male)	28:25	33:44	18:26	19:23	NS	NS	NS
Education, years (mean ± *SD*)	12.53 ± 4.44	10.10 ± 4.75	11.43 ± 4.38	10.27 ± 4.89	.006	NS	NS
Disease duration, months (mean ± *SD*)	n/a	22.49 ± 20.96	25.31 ± 18.94	33.33 ± 16.19	n/a	.028	.040
UPDRS (mean ± *SD*)	2.50 ± 2.12	24.90 ± 10.32	29.67 ± 13.36	28.00 ± 11.52	.003	NS	NS
MMSE (mean ± *SD*)	28.33 ± 1.24	27.04 ± 2.51	26.36 ± 3.26	24.31 ± 2.78	.003	.006	NS

Abbreviations: AP, atypical parkinsonism; HC, healthy controls; MMSE, mini‐mental state examination; MSA, multiple system atrophy; NS, nonsignificant; PD, Parkinson's disease; PSP, progressive supranuclear palsy; UPDRS, unified Parkinson's disease rating scale.

### 3.2 Group differences in neuroimaging measures

Figures S1–S6 depict differences in the three measures between HC and each diagnostic type and between different diagnostic types. In addition, Tables S2–S4 list clusters of the group differences, accompanied by labels of the modified AAL atlas and coordinates of peak voxels. Differences in GM volume were most noticeable in all group comparisons. As compared to HC, GM volume decreased over the basal ganglia, thalamus, cingulate cortex, insula, superior temporal cortex, and cerebellum in all diagnostic types of patients. In addition, reductions in GM volume reached the frontal cortex, sensorimotor cortices, parietal cortex, and occipital cortex in PD and PSP. Between different diagnostic types, reductions in GM volume were more severe primarily over the cerebellum in both MSA and PSP compared to PD, and greater decreases in GM volume were seen over the thalamus, cingulate cortex, and frontal cortex in PSP than in MSA.

Differences in functional measures were also observed but not in all diagnostic types, and group differences were much less distributed than those seen for GM volume. In comparison with HC, decreases in ReHo were observed over the cerebellum in MSA, whereas reductions in DegCen were seen over the cingulate cortex commonly in PD and PSP and over the sensorimotor cortices as well in PD.

### 3.3 Classification accuracy of SVM classifiers

**FIGURE 1:**
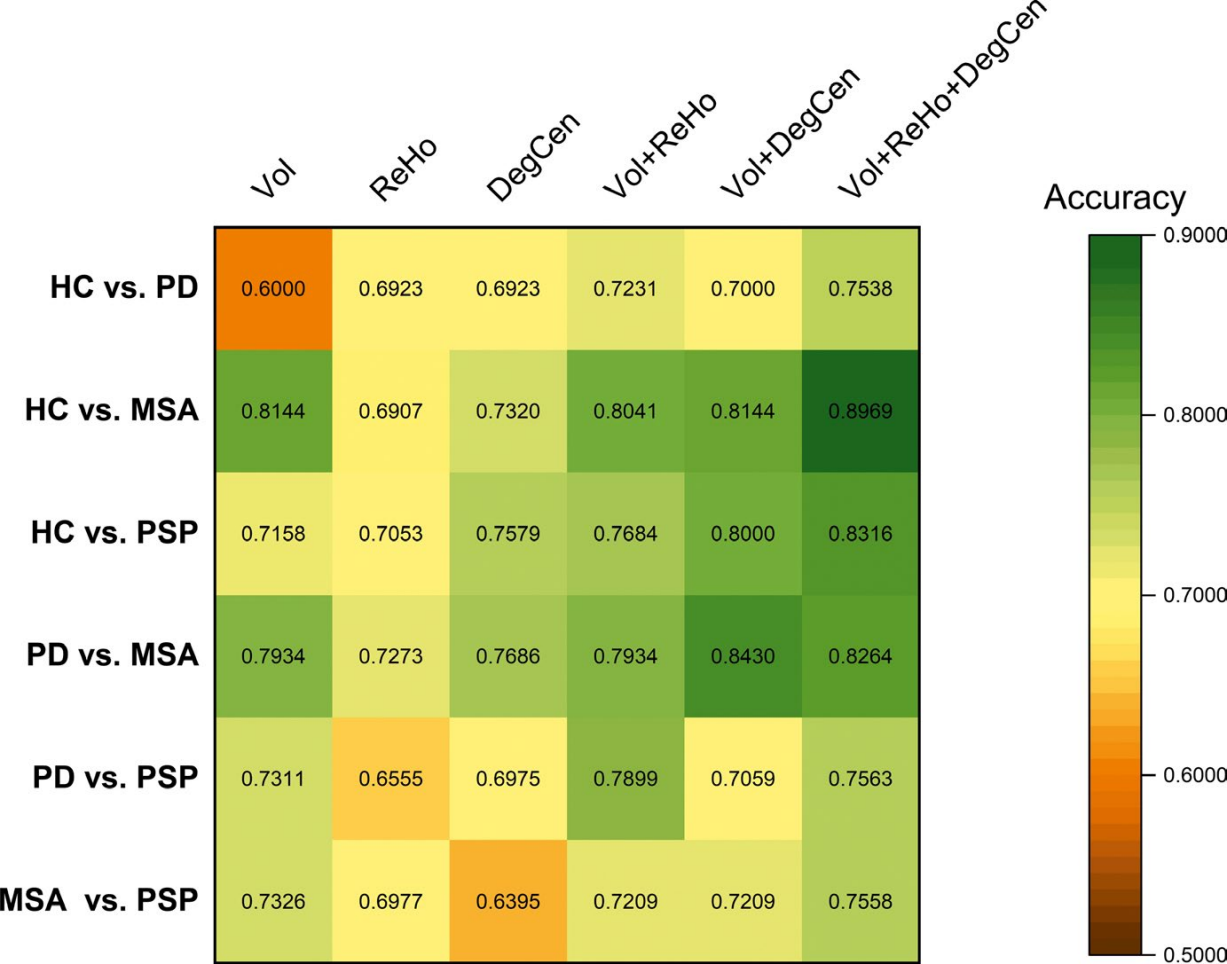
A heat map of the classification accuracy of support vector machine (SVM) classifiers for different classification problems. In the SVM classifiers, individual measures or combinations of those were employed as predictor sets. The considered measures included gray matter volume (Vol), regional homogeneity (ReHo), and degree centrality (DegCen). HC, healthy controls; MSA, multiple system atrophy; PD, Parkinson's disease; PSP, progressive supranuclear palsy

**FIGURE 2:**
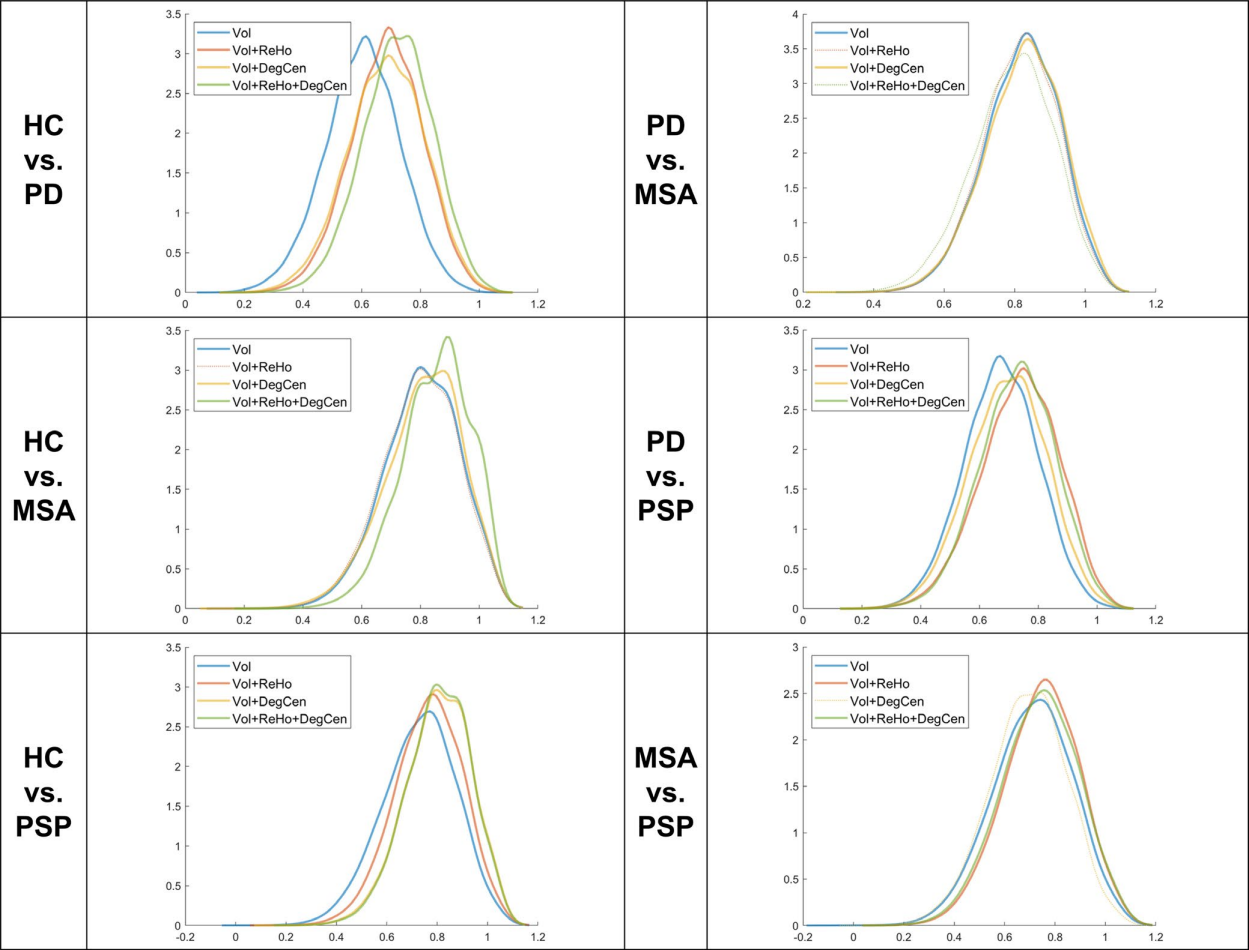
Probability density curves of classification accuracy estimates acquired via 10,000 times of resampling. The probability density curve for the support vector machine (SVM) classifier constructed with gray matter volume (Vol) alone is indicated by a blue solid line. In relation to this SVM classifier, the other probability density curve is indicated in a different color by a solid line when the respective SVM classifier constructed by adding one or more functional measures, among regional homogeneity (ReHo) and degree centrality (DegCen), to Vol has higher classification accuracy of statistical significance or by a dotted line otherwise

A heat map in Figure 1 exhibits variations in the classification accuracy of SVM classifiers according to different combinations of measures. Also, for the SVM classifier constructed with the structural measure alone and those composed by the combination of the structural and functional measures, Figure 2 shows probability density curves of classification accuracy estimates and Table 2 lists statistically significant differences in classification accuracy. In general, predictor sets composed by the combination of the structural and functional measures provided comparable or higher classification accuracy compared to those constructed with a single measure across the different classification problems. The combination of all the three measures yielded the highest classification accuracy in discriminating either diagnostic type from HC and in classifying the two subtypes of AP, with significantly higher classification accuracy than the structural measure alone. In distinguishing PD from MSA or PSP, the combination of GM volume and one functional measure provided the highest classification accuracy, which was significantly higher than that yielded by the structural measure alone. When we assessed classification accuracy by applying the same predictive modeling procedure to the choice of different atlases, the fusion of more than two measures still tended to yield improvements to classification accuracy across the different classification problems, as displayed in Figures S7 and S8.

**TABLE 2:** Statistically significant differences in classification accuracy between support vector machine (SVM) classifiers

	Vol + ReHo	Vol + DegCen	Vol + ReHo + DegCen
HC versus PD	0.083 (*p* value < .001)	0.079 (*p* value < .001)	0.119 (*p* value < .001)
HC versus MSA	NS	0.005 (*p* value < .001)	0.047 (*p* value < .001)
HC versus PSP	0.036 (*p* value < .001)	0.075 (*p* value < .001)	0.076 (*p* value < .001)
PD versus MSA	NS	0.004 (*p* value < .001)	NS
PD versus PSP	0.063 (*p* value < .001)	0.023 (*p* value < .001)	0.055 (*p* value < .001)
MSA versus PSP	0.035 (*p* value < .001)	NS	0.029 (*p* value < .001)

Note

All comparisons were made between the SVM classifier constructed with gray matter volume (Vol) alone and that constructed by adding one or more functional measures, among regional homogeneity (ReHo) and degree centrality (DegCen), to Vol. In case of statistical significance, a mean difference in classification accuracy and its respective *p* value are listed.

Abbreviations: HC, healthy controls; MSA, multiple system atrophy; NS, nonsignificant; PD, Parkinson's disease; PSP, progressive supranuclear palsy.

### 3.4 Contributions of GM regions to classification

**FIGURE 3:**
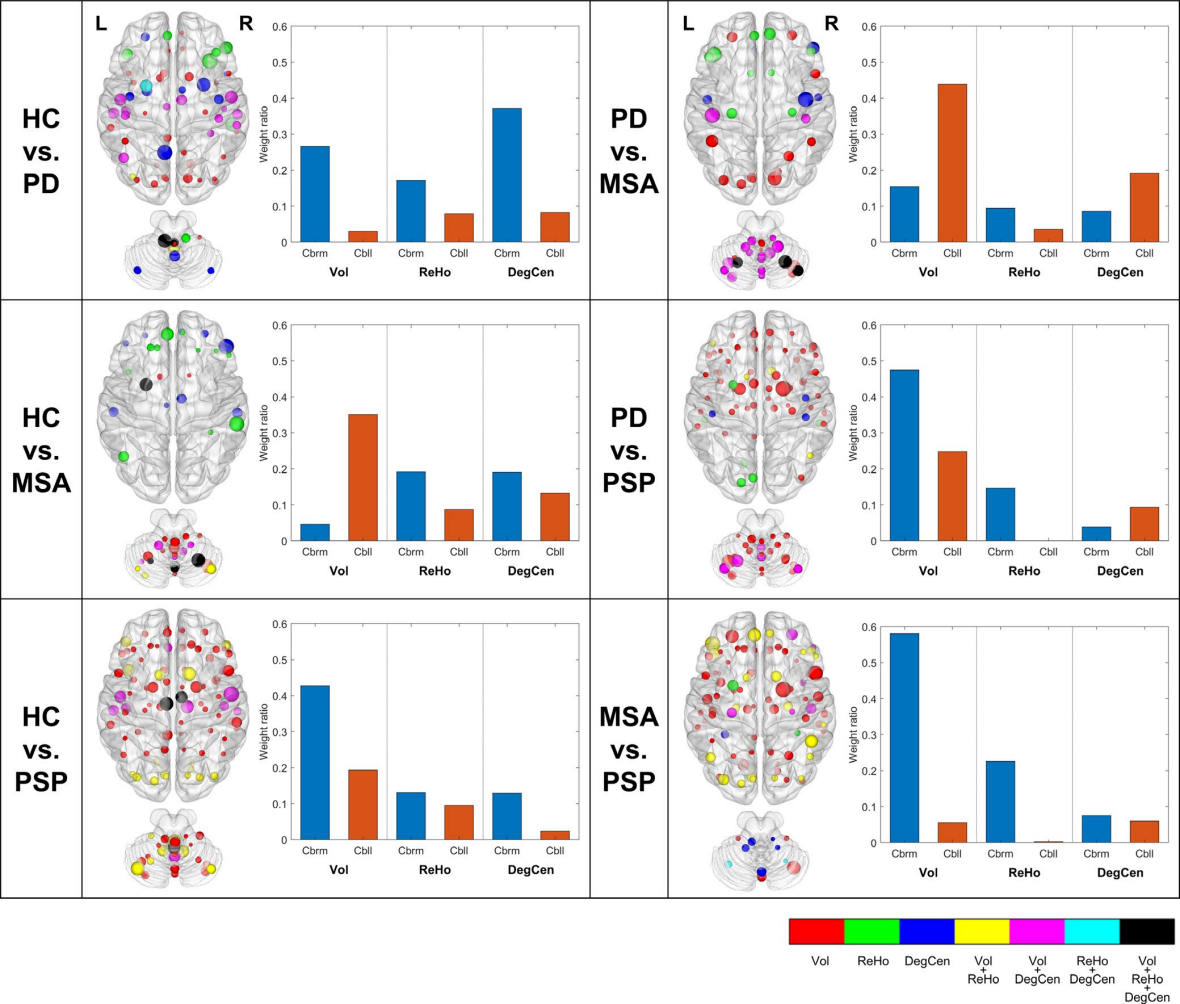
Contributions of gray matter (GM) regions to different classification problems in the support vector machine classifier that has been composed by the combination of three measures. Predictor values were collected from individual GM regions for the three measures, including GM volume (Vol), regional homogeneity (ReHo), and degree centrality (DegCen). The size of a sphere corresponding to each GM region expresses the relative magnitude of its absolute weight, and the color of the sphere indicates the degree of overlaps between the different measures. Inset plots represent relative weight ratios of cerebral (Cbrm) and cerebellar (Cbll) regions according to the different measures

Figure 3 shows predictors in the SVM classifier that has been composed by the combination of all the three measures. GM regions involved in both the structural and functional measures were up to 32% of cerebral regions in distinguishing between MSA and PSP and up to 71% of cerebellar regions in distinguishing between PD and MSA (Table S5). In terms of relative weight ratios of cerebral and cerebellar regions (Figure 3), the relative weight ratios of cerebral regions were generally higher in the classification between HC and PD, between HC and PSP, between PD and PSP, and between MSA and PSP, whereas those of cerebellar regions were largely higher in the classification between HC and MSA and between PD and MSA.

## 4 DISCUSSION

With respect to the diagnosis of PD and AP, there are a range of classification problems, which include distinguishing between PD and AP and between subtypes of AP as well as discriminating PD and AP from HC. In the current investigation, as an effective strategy that can be used to tackle such diverse diagnostic classification problems, we proposed employing multimodal neuroimaging measures in machine learning‐based predictive modeling. We demonstrated that the combined use of the structural and functional measures could improve the performance of SVM classifiers, compared to the use of the structural measure alone, for most of the classification problems even when statistical group differences of the functional measures were not extensively seen.

As machine learning‐based predictive modeling has drawn attention in the era of precision medicine, the potential of multimodal neuroimaging to provide more informative predictors for machine learning has been underlined (Libero, DeRamus, Lahti, Deshpande, & Kana, 2015; Liem et al., 2017; Meng et al., 2017). Structure and function are two main aspects for explaining brain abnormalities, with each conveying a large amount of information. Here, we employed sMRI to acquire a local measure of GM structure and rsfMRI to obtain local and global measures of GM function, and we employed the individual ones or combinations of those as predictor sets for various classification problems.

In agreement with previous studies on structural abnormalities in PD and AP (Brenneis et al., 2003; Messina et al., 2011; Summerfield et al., 2005), we showed that GM volume was reduced in all diagnostic types in relation to HC. Also, as already reported in literature (Messina et al., 2011), more severe GM volume loss in both subtypes of AP than in PD was observed. The general aspects of those structural alterations may be described in terms of differential involvements of cerebral and cerebellar regions: Cerebral regions were mainly affected in PD; cerebellar regions in MSA; and both cerebral and cerebellar regions in PSP.

Similarly, though much more spatially limited, functional alterations affected cerebellar regions in MSA and cerebral regions in PD and PSP, with differential detections of those according to local and global functional measures. Of note, the involvement of the same regions, the sensorimotor cortices and cingulate cortex for PD, the cingulate cortex for PSP, and the cerebellum for MSA, in both structural and functional alterations, reflects a link between structural and functional abnormalities.

When the structural and functional measures were employed as predictor sets for discriminating each diagnostic type from HC and distinguishing between different diagnostic types, statistical group differences were not always directly connected with the performance of SVM classifiers. For instance, although GM volume was the measure of the most prominent differences between HC and PD at a group level, it yielded lower classification performance than the functional measures. Nevertheless, when we combined the structural and functional measures in constructing predictor sets, classification performance became generally enhanced, and particularly, the combination of all the three measures yielded significantly higher classification accuracy than the structural measure alone in all classification problems but distinguishing between PD and MSA. As it is already recognized in clinical practice that multiple diagnostic criteria help to increase the level of diagnostic accuracy (Irene Litvan, 1999), the addition of the functional measures seems to yield enhanced classification performance by providing additional diagnostic criteria possibly based on the relevance of functional abnormalities to the pathophysiology of PD and AP.

Since, from a pathogenic perspective, neuronal degeneration constitutes pathologic lesions, structural abnormalities seem to be predominant in PD and AP (Eriksen, Wszolek, & Petrucelli, 2005), and in the current investigation, this was evident in statistical comparisons of the structural and functional measures. Nonetheless, it would be obvious as well that not all aspects of the distinction between groups can be explained by structural alterations alone even when functional alterations are not clearly revealed in statistical comparisons at a group level. As shown in Figure 3, the involvement of a substantial portion of GM regions, including the sensorimotor cortices, cingulate cortex, and cerebellum mentioned above, in predictors of both structural and functional measures indicates again a link between structural and functional abnormalities. Moreover, local and global functional measures had different contributions to classification, so that the inclusion of both functional measures would be largely helpful in enhancing classification performance.

With respect to the notion that multimodal neuroimaging measures can be fused to generally improve performance for various prediction problems, it might be tempted to add as wide a variety of measures as available. However, it is also important to understand that improvements to prediction performance can be not always guaranteed by the combined use of multimodal neuroimaging measures (Schmaal et al., 2015). In this regard, requirements for enhanced prediction performance may be mentioned in two aspects. First, informative measures that can characterize the pathogenesis or pathophysiology of a disease need to constitute predictors for machine learning (Deo, 2015). As additional informative MRI measures, for instance, white matter structural measures that can be acquired from diffusion weighted MRI would be promising, as they can reveal pathological correlates of parkinsonism (Quattrone et al., 2006; Scherfler, 2005; Schocke et al., 2002). Second, having collected a large number of measures, a crucial process for composing a predictor set is feature reduction, which involves selecting important measures or combining existing measures. Here, we used the filter method as a simple way of feature reduction, but a more advanced approach to feature reduction could be considered (Meng et al., 2017). Alternatively, deep learning may be employed since it provides the capability of data‐driven automatic feature generation (Arbabshirani, Plis, Sui, & Calhoun, 2017).

This study has limitations to consider. For each diagnostic type, although clinical diagnoses were finally made after more than three years of a clinical follow‐up, the possibility of misdiagnosis cannot be ruled out due to a lack of pathological confirmation. In addition, as regards our machine learning approach, the sample size in this study was small, and moreover, the generalization ability of the predictive models was not fully evaluated in that we performed internal cross‐validation only. Further investigation including an external validation procedure is warranted in the future. Besides the diagnostic classification problems we considered, the differential diagnosis of two phenotypes of MSA, MSA‐P, and MSA‐C, would be of clinical interest as well (Garraux et al., 2013). Although structural and functional abnormalities may be specific to individual MSA variants (Planetta et al., 2015), we merged the two phenotypes of MSA as a single group here primarily due to a possible overlap of clinical and pathological findings between the two (Krismer et al., 2019; Wenning et al., 2013) in relation to our limited sample size of each.

In conclusion, we demonstrated that the fusion of different measures from multimodal neuroimaging would have potential for improving the performance of machine learning‐based predictive models. For PD and AP, although functional alterations are much more limited than structural alterations, a possible link between structural and functional abnormalities appears to support the informativeness of functional measures, and the combined use of structural and functional measures is likely to yield improvements to performance in various diagnostic classification problems. In machine learning approaches to predictive modeling for clinical concerns, the limited sample size has been a major obstacle (Sakai & Yamada, 2019). Although it may be often unsure which neuroimaging measures would be suitable for a specific prediction problem, we propose that, in addition to increasing the number of patients, gathering diverse informative measures from multimodal neuroimaging for each patient would be helpful to develop a better performing predictive model. In addition, for the clinical use of multimodal neuroimaging measures, although the computational time to process multimodal neuroimaging data, rather than their acquisition time, may be a potential limitation, it could be efficiently managed by taking advantage of recent technical advances toward the automated and intelligent processing of multimodal neuroimaging data.

## CONFLICT OF INTEREST

The authors have nothing to disclose.

## AUTHOR CONTRIBUTION

SJC and N‐YS were responsible for the study concept and design. PHL and S‐KL contributed to the collection of data. CP analyzed the data and drafted the manuscript. All authors critically reviewed content and approved final version for publication.

### Peer Review

The peer review history for this article is available at https://publons.com/publon/10.1002/brb3.1808.

## Supporting information

Appendix S1

## Data Availability

The data that support the findings of this study are available from the corresponding author upon reasonable request.
