# Supplementary material for: The diagnostic potential of multimodal neuroimaging measures in Parkinson's disease and atypical parkinsonism
Source: Brain Behav. 2020 Oct 7;10(11):e01808. doi: 10.1002/brb3.1808 (PMC7667347; doi:10.1002/brb3.1808)
Supplement: Supplementary file 1 — Appendix S1 [file BRB3-10-e01808-s001.pdf]

Supporting Information:

The diagnostic potential of multimodal neuroimaging measures in Parkinson's disease and atypical parkinsonism

Chang-hyun Park[1,2], Phil Hyu Lee[3], Seung-Koo Lee[4], Seok Jong Chung[3,5], Na-Young Shin[1]

[1]Department of Radiology, Catholic University of Korea College of Medicine, Seoul, Kore
[2]Center for Neuroprosthetics and Brain Mind Institute, Swiss Federal Institute of Technology (EPFL), Geneva, Switzerland
[3]Department of Neurology, Yonsei University College of Medicine, Seoul, Korea
[4]Department of Radiology, Yonsei University College of Medicine, Seoul, Korea
[5]Department of Neurology, Yongin Severance Hospital, Yonsei University Health System, Yongin, Korea

Supporting Methods

Processing of neuroimaging data

Using the tools in SPM12 (http://www.fil.ion.ucl.ac.uk/spm/, RRID:SCR_007037), sMRI data were segmented into different brain tissues, including grey matter (GM), white matter (WM), and corticospinal fluid. The GM segmentation map was spatially transformed to the standard space and it was modulated so that the total amount of GM remained the same as in the original one in the native space. The normalized and modulated map represented voxel-wise volume of GM and it was smoothed with a Gaussian kernel of 8 mm full width at half maximum.

Using the tools in SPM12 and DPARSF (http://rfmri.org/DPARSF/, RRID:SCR_002372), rsfMRI data were spatially realigned to correct for head movement and they were spatially transformed to the standard space. In addition, linear detrending was applied to remove systematic signal increases or decreases, nuisance covariates regression was applied to reduce effects of head movement and non-neuronal fluctuations, and band-pass filtering at 0.01 - 0.08 Hz was applied to reduce effects of very low and high frequencies physiological noise. The preprocessed rsfMRI data were employed to measure voxel-wise regional homogeneity (ReHo) and degree centrality (DegCen). ReHo was assessed by computing Kendall's coefficient of concordance (KCC) in time series of neighboring voxels (Zang, Jiang, Lu, He, & Tian, 2004), such that a KCC value represented the degree of temporal similarity between neighboring voxels. DegCen was assessed by computing the number of connections incident upon a voxel when a connection was regarded to be present if the correlation coefficient in time series of a pair of voxels was greater than 0.25 (Lee et al., 2014). The ReHo and DegCen maps were smoothed with a Gaussian kernel of 8 mm full width at half maximum.

Acquisition of prediction values according to different atlas selections

In addition to the modified automated anatomical labeling (AAL) atlas (Rolls, Joliot, & Tzourio-Mazoyer, 2015) that contains parcellations of 120 GM regions, we also considered the original AAL atlas (Tzourio-Mazoyer et al., 2002) and Hammers atlas (Hammers et al., 2003). The AAL atlas includes 90 cerebral regions and 26 cerebellar regions, and the Hammers atlas includes 58 cerebral regions and 2 cerebellar regions. Similarly to 120 GM regions based on the modified AAL atlas, predictor values were acquired for 116 GM regions based on the AAL atlas and for 60 GM regions based on the Hammers atlas. That is, voxel-wise values of GM volume, ReHo, and DegCen were averaged over the voxels of each GM region, and the averaged value was directly assigned to the respective GM region for ReHo and DegCen, whereas its ratio to total intracranial volume was assigned to the respective GM region for GM volume.

Supporting Tables and Figures

Table S1. The list of 120 grey matter regions the parcellation of which was determined according to the modified automated anatomical labeling atlas (Rolls et al., 2015).

Index	Region name	Anatomical subdivision
1	Left precental gyrus	Central region
2	Right precental gyrus	Central region
3	Left superior frontal gyrus, dorsolateral	Frontal lobe - lateral surface
4	Right superior frontal gyrus, dorsolateral	Frontal lobe - lateral surface
5	Left middle frontal gyrus	Frontal lobe - lateral surface
6	Right middle frontal gyrus	Frontal lobe - lateral surface
7	Left inferior frontal gyrus, opercular	Frontal lobe - lateral surface
8	Right inferior frontal gyrus, opercular	Frontal lobe - lateral surface
9	Left inferior frontal gyrus, triangular	Frontal lobe - lateral surface
10	Right inferior frontal gyrus, triangular	Frontal lobe - lateral surface
11	Left inferior frontal gyrus, orbital	Frontal lobe - orbital surface
12	Right inferior frontal gyrus, orbital	Frontal lobe - orbital surface
13	Left rolandic operculum	Central region
14	Right rolandic operculum	Central region
15	Left supplementary motor area	Frontal lobe - medial surface
16	Right supplementary motor area	Frontal lobe - medial surface
17	Left olfactory cortex	Frontal lobe - orbital surface
18	Right olfactory cortex	Frontal lobe - orbital surface
19	Left superior frontal gyrus, medial	Frontal lobe - medial surface
20	Right superior frontal gyrus, medial	Frontal lobe - medial surface
21	Left superior frontal gyrus, medial orbital	Frontal lobe - orbital surface
22	Right superior frontal gyrus, medial orbital	Frontal lobe - orbital surface
23	Left gyrus rectus	Frontal lobe - orbital surface
24	Right gyrus rectus	Frontal lobe - orbital surface
25	Left medial orbitral gyrus	Frontal lobe - orbital surface
26	Right medial orbitral gyrus	Frontal lobe - orbital surface
27	Left anterior orbitral gyrus	Frontal lobe - orbital surface
28	Right anterior orbitral gyrus	Frontal lobe - orbital surface
29	Left posterior orbitral gyrus	Frontal lobe - orbital surface
30	Right posterior orbitral gyrus	Frontal lobe - orbital surface
31	Left lateral orbitral gyrus	Frontal lobe - orbital surface
32	Right lateral orbitral gyrus	Frontal lobe - orbital surface
33	Left insula	Limbic lobe
34	Right insula	Limbic lobe
35	Left anterior cingulate gyrus	Limbic lobe
36	Right anterior cingulate gyrus	Limbic lobe
37	Left middle cingulate gyrus	Limbic lobe

38	Right middle cingulate gyrus	Limbic lobe
39	Left posterior cingulate gyrus	Limbic lobe
40	Right posterior cingulate gyrus	Limbic lobe
41	Left hippocampus	Limbic lobe
42	Right hippocampus	Limbic lobe
43	Left parahippocampal gyrus	Limbic lobe
44	Right parahippocampal gyrus	Limbic lobe
45	Left amygdala	Subcortical grey nuclei
46	Right amygdala	Subcortical grey nuclei
47	Left calcarine sulcus	Occipital lobe - medial/inferior surfaces
48	Right calcarine sulcus	Occipital lobe - medial/inferior surfaces
49	Left cuneus	Occipital lobe - medial/inferior surfaces
50	Right cuneus	Occipital lobe - medial/inferior surfaces
51	Left lingual gyrus	Occipital lobe - medial/inferior surfaces
52	Right lingual gyrus	Occipital lobe - medial/inferior surfaces
53	Left superior occipital gyrus	Occipital lobe - lateral surface
54	Right superior occipital gyrus	Occipital lobe - lateral surface
55	Left middle occipital gyrus	Occipital lobe - lateral surface
56	Right middle occipital gyrus	Occipital lobe - lateral surface
57	Left inferior occipital gyrus	Occipital lobe - lateral surface
58	Right inferior occipital gyrus	Occipital lobe - lateral surface
59	Left fusiform gyrus	Occipital lobe - medial/inferior surfaces
60	Right fusiform gyrus	Occipital lobe - medial/inferior surfaces
61	Left postcentral gyrus	Central region
62	Right postcentral gyrus	Central region
63	Left superior parietal gyrus	Parietal lobe - lateral surface
64	Right superior parietal gyrus	Parietal lobe - lateral surface
65	Left inferior parietal gyrus	Parietal lobe - lateral surface
66	Right inferior parietal gyrus	Parietal lobe - lateral surface
67	Left supramarginal gyrus	Parietal lobe - lateral surface
68	Right supramarginal gyrus	Parietal lobe - lateral surface
69	Left angular gyrus	Parietal lobe - lateral surface
70	Right angular gyrus	Parietal lobe - lateral surface
71	Left precuneus	Parietal lobe - medial surface
72	Right precuneus	Parietal lobe - medial surface
73	Left paracentral lobule	Frontal lobe - medial surface
74	Right paracentral lobule	Frontal lobe - medial surface
75	Left caudate nucleus	Subcortical grey nuclei
76	Right caudate nucleus	Subcortical grey nuclei
77	Left lenticular nucleus, putamen	Subcortical grey nuclei
78	Right lenticular nucleus, putamen	Subcortical grey nuclei
79	Left lenticular nucleus, globas pallidus	Subcortical grey nuclei
80	Right lenticular nucleus, globus pallidus	Subcortical grey nuclei

81	Left thalamus	Subcortical grey nuclei
82	Right thalamus	Subcortical grey nuclei
83	Left transverse temporal gyrus	Temporal lobe - lateral surface
84	Right transverse temporal gyrus	Temporal lobe - lateral surface
85	Left superior temporal gyrus	Temporal lobe - lateral surface
86	Right superior temporal gyrus	Temporal lobe - lateral surface
87	Left superior temporal pole	Limbic lobe
88	Right superior temporal pole	Limbic lobe
89	Left middle temporal gyrus	Temporal lobe - lateral surface
90	Right middle temporal gyrus	Temporal lobe - lateral surface
91	Left middle temporal pole	Limbic lobe
92	Right middle temporal pole	Limbic lobe
93	Left inferior temporal gyrus	Temporal lobe - lateral surface
94	Right inferior temporal gyrus	Temporal lobe - lateral surface
95	Left crus I	Cerebellum - hemisphere
96	Right crus I	Cerebellum - hemisphere
97	Left crus II	Cerebellum - hemisphere
98	Right crus II	Cerebellum - hemisphere
99	Left lobule III	Cerebellum - hemisphere
100	Right lobule III	Cerebellum - hemisphere
101	Right lobule IV, V	Cerebellum - hemisphere
102	Left lobule IV, V	Cerebellum - hemisphere
103	Left lobule VI	Cerebellum - hemisphere
104	Right lobule VI	Cerebellum - hemisphere
105	Left lobule VIIB	Cerebellum - hemisphere
106	Right lobule VIIB	Cerebellum - hemisphere
107	Left lobule VIII	Cerebellum - hemisphere
108	Right lobule VIII	Cerebellum - hemisphere
109	Right lobule IX	Cerebellum - hemisphere
110	Left lobule IX	Cerebellum - hemisphere
111	Left lobule X	Cerebellum - hemisphere
112	Right lobule X	Cerebellum - hemisphere
113	Lobule I, II of vermis	Cerebellum - Vermis
114	Lobule III of vermis	Cerebellum - Vermis
115	Lobule IV, V of vermis	Cerebellum - Vermis
116	Lobule VI of vermis	Cerebellum - Vermis
117	Lobule VII of vermis	Cerebellum - Vermis
118	Lobule VIII of vermis	Cerebellum - Vermis
119	Lobule IX of vermis	Cerebellum - Vermis
120	Lobule X of vermis	Cerebellum - Vermis

Table S2. Areas of differences in gray matter volume between groups, corresponding to Figures S1 and S2.

(A) Healthy controls (HC) vs. Parkinson's disease (PD)

**Contrast**	**Cluster**	**Cluster size**	**Area**	***t* value**	***p* value**	**Coordinates (mm)**		
						x	***y***	***z***
	1	9979	Left calcarine sulcus	5.5334	< 0.0001	-4	-70	12
			Right lingual gyrus	5.2815	< 0.0001	14	-66	-6
			Right angular gyrus	4.9306	< 0.0001	50	-62	32
			Left fusiform gyrus	4.8217	< 0.0001	-24	-82	-18
			Right calcarine sulcus	4.8057	< 0.0001	2	-76	8
			Left middle cingulate gyrus	4.7947	< 0.0001	-8	-18	36
			Right cuneus	4.7894	< 0.0001	6	-72	22
			Right precuneus	4.7053	< 0.0001	4	-70	22
			Left posterior cingulate gyrus	4.6900	< 0.0001	-6	-46	32
			Right supramarginal gyrus	4.6810	< 0.0001	58	-28	34
			Left lingual gyrus	4.6770	< 0.0001	-22	-82	-16
			Left precuneus	4.6551	< 0.0001	-6	-50	34
			Right medial orbitral gyrus	4.6447	< 0.0001	26	30	-18
			Right superior temporal gyrus	4.6397	< 0.0001	60	-10	6
			Left cuneus	4.6152	< 0.0001	4	-82	20
			Right middle cingulate gyrus	4.5563	< 0.0001	8	-38	34
			Right transverse temporal gyrus	4.5554	< 0.0001	60	-8	6
			Left crus I	4.5219	< 0.0001	-26	-82	-20
			Right superior parietal gyrus	4.5034	< 0.0001	20	-68	50
			Right posterior orbitral gyrus	4.4520	< 0.0001	26	28	-20
			Right rolandic operculum	4.4261	< 0.0001	60	-8	8
			Right insula	4.4172	< 0.0001	38	-2	10
			Left lobule VI	4.3731	< 0.0001	-26	-80	-20
			Right precental gyrus	4.3512	< 0.0001	52	2	26
			Right middle temporal gyrus	4.3501	< 0.0001	60	-46	10
			Right inferior parietal gyrus	4.3346	< 0.0001	52	-46	40
HC > PD			Lobule VI of vermis	4.3021	< 0.0001	4	-70	-6
			Right olfactory cortex	4.3009	< 0.0001	24	12	-18
			Right fusiform gyrus	4.2327	< 0.0001	24	-74	-12
			Right inferior frontal gyrus, opercular	4.1163	< 0.0001	46	8	26
			Right anterior orbitral gyrus	4.1099	< 0.0001	26	34	-16
			Right middle occipital gyrus	4.0979	< 0.0001	28	-80	16
			Right superior occipital gyrus	4.0930	< 0.0001	28	-80	18
			Right inferior occipital gyrus	4.0688	< 0.0001	38	-84	-6
			Right postcentral gyrus	4.0009	0.0001	56	-8	30
			Right superior temporal pole	3.9785	0.0001	52	4	-2
			Right inferior frontal gyrus, orbital	3.9729	0.0001	36	24	-10
			Right anterior cingulate gyrus	3.8367	0.0001	6	30	24
			Right inferior temporal gyrus	3.7815	0.0001	46	-70	-6
	2	6089	Left superior temporal gyrus	5.6724	< 0.0001	-56	-22	12
			Left transverse temporal gyrus	5.6031	< 0.0001	-46	-20	10
			Left supramarginal gyrus	5.5380	< 0.0001	-58	-38	30
			Left rolandic operculum	5.4023	< 0.0001	-44	-20	12
			Left postcentral gyrus	5.3611	< 0.0001	-52	-14	28
			Left insula	5.3583	< 0.0001	-38	18	4
			Left inferior parietal gyrus	5.3223	< 0.0001	-44	-28	44
			Left inferior frontal gyrus, triangular	5.2242	< 0.0001	-40	18	6
			Left middle temporal gyrus	5.1543	< 0.0001	-60	-38	-6
			Left inferior frontal gyrus, opercular	5.0161	< 0.0001	-46	10	22
			Left angular gyrus	4.7517	< 0.0001	-50	-60	24
			Left middle occipital gyrus	4.6029	< 0.0001	-46	-72	16
			Left precental gyrus	4.5801	< 0.0001	-54	-4	24
			Left superior temporal pole	4.5437	< 0.0001	-52	4	0

			Left lenticular nucleus, putamen	4.3563	< 0.0001	-24	2	-10
			Left posterior orbitral gyrus	4.2710	< 0.0001	-22	10	-18
			Left middle frontal gyrus	4.2601	< 0.0001	-40	8	36
			Left olfactory cortex	4.2248	< 0.0001	-20	10	-18
			Left inferior frontal gyrus, orbital	4.1896	< 0.0001	-36	22	-6
			Left inferior occipital gyrus	4.1154	< 0.0001	-44	-76	-4
			Left medial orbitral gyrus	3.9349	0.0001	-14	14	-20
			Left amygdala	3.8705	0.0001	-18	0	-12
	3	1893	Right hippocampus	4.7396	< 0.0001	26	-14	-12
			Left caudate nucleus	4.1009	< 0.0001	-10	12	0
			Right superior frontal gyrus, medial orbital	4.0671	< 0.0001	10	44	-2
			Right caudate nucleus	4.0612	< 0.0001	14	2	18
			Right superior frontal gyrus, medial	3.9572	0.0001	10	44	0
			Right lingual gyrus	3.8161	0.0001	18	-28	-10
			Right anterior cingulate gyrus	3.8016	0.0001	10	46	2
			Right parahippocampal gyrus	3.7945	0.0001	20	-28	-12
			Right thalamus	3.7812	0.0001	14	-34	6
			Left anterior cingulate gyrus	3.7396	0.0001	2	20	-8
			Right olfactory cortex	3.6673	0.0002	2	20	-6
			Left olfactory cortex	3.6199	0.0002	-2	18	-6
	4	1017	Left middle frontal gyrus	5.5634	< 0.0001	-26	44	26
			Left superior frontal gyrus, dorsolateral	5.4579	< 0.0001	-26	46	24
			Left superior frontal gyrus, medial	4.4000	< 0.0001	-6	42	38
			Left inferior frontal gyrus, triangular	4.0135	0.0001	-38	36	24
	5	311	Left lingual gyrus	3.8822	0.0001	-26	-44	-10
			Left parahippocampal gyrus	3.8384	0.0001	-26	-42	-10
			Left hippocampus	3.8231	0.0001	-12	-36	6
			Left fusiform gyrus	3.8085	0.0001	-26	-42	-12
			Left thalamus	3.5763	0.0002	-12	-34	6

(B) HC vs. multiple system atrophy (MSA)

**Contrast**	**Cluster**	**Cluster size**	**Area**	***t* value**	***p* value**	**Coordinates (mm)**		
						x	***y***	***z***
HC > MSA	1	25711	Lobule VIII of vermis	7.4598	< 0.0001	6	-66	-42
			Right lobule IX	7.4560	< 0.0001	0	-56	-46
			Left lobule IX	7.4162	< 0.0001	2	-60	-44
			Left lobule VIII	7.3979	< 0.0001	-4	-70	-40
			Lobule IX of vermis	7.3724	< 0.0001	0	-58	-44
			Right lobule VIII	7.3475	< 0.0001	6	-64	-44
			Right crus I	7.2389	< 0.0001	28	-80	-26
			Lobule I, II of vermis	7.1267	< 0.0001	-2	-38	-18
			Right lobule VIIB	7.0860	< 0.0001	34	-70	-50
			Right lobule VI	7.0306	< 0.0001	36	-50	-30
			Lobule III of vermis	6.9788	< 0.0001	-2	-38	-16
			Left lobule III	6.9262	< 0.0001	-4	-38	-18
			Left lobule VI	6.9081	< 0.0001	-4	-62	-12
			Left lobule VIIB	6.8697	< 0.0001	-6	-72	-40
			Right fusiform gyrus	6.7653	< 0.0001	26	-52	-16
			Lobule IV, V of vermis	6.7624	< 0.0001	-2	-62	-12
			Lobule VI of vermis	6.7544	< 0.0001	-2	-64	-12
			Left crus II	6.7433	< 0.0001	-18	-82	-32
			Left crus I	6.6280	< 0.0001	-20	-82	-32
			Right lobule IV, V	6.5408	< 0.0001	-4	-62	-14
			Right crus II	6.4881	< 0.0001	34	-72	-46
			Left insula	6.4431	< 0.0001	-42	-2	6
			Left lobule IV, V	6.3466	< 0.0001	10	-60	-12
			Left fusiform gyrus	6.1972	< 0.0001	-28	-46	-18
			Right lingual gyrus	6.1445	< 0.0001	10	-64	-12
			Lobule X of vermis	6.1435	< 0.0001	-4	-44	-40

			Left anterior cingulate gyrus	6.0515	< 0.0001	0	20	-10
			Left lingual gyrus	6.0400	< 0.0001	-16	-82	-14
			Left olfactory cortex	6.0117	< 0.0001	0	18	-10
			Right lobule III	5.9957	< 0.0001	8	-36	-16
			Right insula	5.9851	< 0.0001	40	-8	6
			Left rolandic operculum	5.8781	< 0.0001	-42	-6	8
			Lobule VII of vermis	5.5934	< 0.0001	-2	-72	-32
			Left superior temporal gyrus	5.5481	< 0.0001	-40	-18	0
			Right superior frontal gyrus, medial orbital	5.4804	< 0.0001	0	22	-10
			Right rolandic operculum	5.4727	< 0.0001	40	-6	10
			Right superior temporal gyrus	5.4614	< 0.0001	44	-14	-2
			Right lobule X	5.2915	< 0.0001	22	-40	-46
			Left transverse temporal gyrus	5.2737	< 0.0001	-40	-20	2
			Right olfactory cortex	5.2040	< 0.0001	2	18	-8
			Left posterior orbitral gyrus	5.1928	< 0.0001	-22	10	-18
			Left parahippocampal gyrus	4.9974	< 0.0001	-18	-18	-24
			Right anterior cingulate gyrus	4.9451	< 0.0001	2	22	-8
			Right parahippocampal gyrus	4.8110	< 0.0001	20	-20	-24
			Right hippocampus	4.8020	< 0.0001	24	-14	-20
			Right posterior orbitral gyrus	4.7631	< 0.0001	22	14	-20
			Right amygdala	4.7407	< 0.0001	24	6	-16
			Right middle temporal gyrus	4.6416	< 0.0001	62	-36	6
			Left superior temporal pole	4.5928	< 0.0001	-44	0	-20
			Left thalamus	4.5859	< 0.0001	-2	-12	8
			Left hippocampus	4.5480	< 0.0001	-22	-16	-22
			Left lobule X	4.5470	< 0.0001	-14	-38	-46
			Left caudate nucleus	4.5316	< 0.0001	-10	18	2
			Right medial orbitral gyrus	4.4884	< 0.0001	20	14	-20
			Left middle temporal gyrus	4.4497	< 0.0001	-44	-2	-18
			Right superior temporal pole	4.3411	< 0.0001	44	2	-14
			Right transverse temporal gyrus	4.2662	< 0.0001	42	-16	6
			Left precentral gyrus	4.2639	< 0.0001	-52	6	16
			Left inferior frontal gyrus, orbital	4.2558	< 0.0001	-34	24	-6
			Right superior frontal gyrus, medial	4.2049	< 0.0001	8	46	0
			Left inferior frontal gyrus, triangular	4.1902	< 0.0001	-46	12	24
			Left medial orbitral gyrus	4.1889	< 0.0001	-16	12	-20
			Right lenticular nucleus, putamen	4.1774	< 0.0001	24	10	-4
			Right caudate nucleus	4.1365	< 0.0001	10	6	14
			Right thalamus	4.1253	< 0.0001	2	-14	8
			Left lenticular nucleus, putamen	4.1249	< 0.0001	-24	8	-2
			Right inferior frontal gyrus, orbital	4.1061	< 0.0001	34	24	-12
			Left inferior frontal gyrus, opercular	4.0701	< 0.0001	-46	12	22
			Left postcentral gyrus	3.8358	0.0001	-58	-12	14
			Left amygdala	3.7812	0.0001	-24	4	-18
			Right inferior occipital gyrus	3.7165	0.0002	32	-84	-10

(C) HC vs. progressive supranuclear palsy (PSP)

**Contrast**	**Cluster**	**Cluster size**	**Area**	***t* value**	***p* value**	**Coordinates (mm)**		
						x	***y***	***z***
HC > PSP	1	49886	Left thalamus	9.6405	< 0.0001	-2	-14	8
			Right thalamus	8.9403	< 0.0001	2	-14	8
			Lobule III of vermis	8.4307	< 0.0001	-2	-38	-12
			Left lobule III	8.3866	< 0.0001	-4	-38	-10
			Left insula	7.9484	< 0.0001	-38	18	4
			Left hippocampus	7.6331	< 0.0001	-14	-36	0
			Right caudate nucleus	7.5831	< 0.0001	12	2	18
			Left lobule VI	7.5422	< 0.0001	-10	-68	-14
			Right lobule IV, V	7.5260	< 0.0001	-6	-36	-8
			Left superior temporal gyrus	7.4047	< 0.0001	-42	-16	-4

Left lingual gyrus	7.4038	< 0.0001	-10	-34	-4
Right insula	7.3227	< 0.0001	42	-12	2
Lobule I, II of vermis	7.2144	< 0.0001	0	-38	-18
Left inferior frontal gyrus, triangular	7.1210	< 0.0001	-40	22	4
Right hippocampus	7.0595	< 0.0001	18	-34	2
Right precuneus	7.0555	< 0.0001	14	-36	2
Left precuneus	7.0409	< 0.0001	-12	-38	2
Right rolandic operculum	7.0123	< 0.0001	42	-12	16
Right superior temporal gyrus	6.8742	< 0.0001	44	-14	0
Left rolandic operculum	6.7931	< 0.0001	-40	-6	10
Left olfactory cortex	6.7862	< 0.0001	0	16	-10
Left gyrus rectus	6.6885	< 0.0001	-4	40	-20
Left parahippocampal gyrus	6.6594	< 0.0001	-16	-36	-4
Right lingual gyrus	6.6140	< 0.0001	10	-34	0
Left inferior frontal gyrus, orbital	6.6043	< 0.0001	-34	24	-6
Left middle cingulate gyrus	6.5619	< 0.0001	-2	-38	34
Left anterior cingulate gyrus	6.4819	< 0.0001	2	18	-10
Left transverse temporal gyrus	6.4060	< 0.0001	-40	-20	2
Left medial orbitral gyrus	6.3622	< 0.0001	-12	12	-20
Left caudate nucleus	6.3418	< 0.0001	-12	-8	20
Right olfactory cortex	6.3218	< 0.0001	2	16	-8
Right lobule IX	6.3124	< 0.0001	-4	-46	-40
Left calcarine sulcus	6.3121	< 0.0001	2	-78	10
Right anterior cingulate gyrus	6.2480	< 0.0001	6	42	-2
Left superior frontal gyrus, medial orbital	6.2233	< 0.0001	-4	44	-8
Lobule VI of vermis	6.2233	< 0.0001	-2	-68	-14
Left posterior cingulate gyrus	6.1875	< 0.0001	0	-38	32
Left superior temporal pole	6.1628	< 0.0001	-26	10	-30
Right superior frontal gyrus, medial orbital	6.1334	< 0.0001	6	44	-2
Right inferior frontal gyrus, opercular	6.1146	< 0.0001	40	16	4
Right transverse temporal gyrus	6.0986	< 0.0001	42	-16	6
Right middle cingulate gyrus	6.0802	< 0.0001	2	-22	38
Right gyrus rectus	6.0569	< 0.0001	2	40	-20
Right calcarine sulcus	6.0307	< 0.0001	12	-60	10
Left lobule IV, V	6.0276	< 0.0001	12	-62	-10
Left lobule VIII	6.0266	< 0.0001	-8	-66	-46
Right inferior frontal gyrus, orbital	6.0182	< 0.0001	36	22	-10
Left inferior frontal gyrus, opercular	5.9872	< 0.0001	-50	10	2
Right lobule VI	5.9723	< 0.0001	8	-66	-10
Right parahippocampal gyrus	5.9637	< 0.0001	20	-22	-16
Left superior frontal gyrus, medial	5.8891	< 0.0001	-6	38	30
Lobule X of vermis	5.8727	< 0.0001	0	-46	-38
Right superior frontal gyrus, medial	5.8682	< 0.0001	8	44	0
Right posterior cingulate gyrus	5.8521	< 0.0001	12	-36	6
Right lobule X	5.8315	< 0.0001	26	-38	-44
Lobule IX of vermis	5.7682	< 0.0001	0	-48	-38
Right lobule III	5.7561	< 0.0001	8	-36	-14
Left middle temporal gyrus	5.7553	< 0.0001	-52	-28	-8
Right fusiform gyrus	5.7264	< 0.0001	36	-44	-24
Right crus I	5.6835	< 0.0001	42	-46	-30
Left lobule VIIB	5.6647	< 0.0001	-6	-72	-40
Left lobule IX	5.5870	< 0.0001	4	-46	-38
Right inferior temporal gyrus	5.5687	< 0.0001	44	-48	-26
Left middle temporal pole	5.5684	< 0.0001	-28	10	-34
Left middle frontal gyrus	5.5406	< 0.0001	-36	52	16
Right inferior frontal gyrus, triangular	5.4727	< 0.0001	46	18	2
Right posterior orbitral gyrus	5.4509	< 0.0001	28	12	-22
Lobule IV, V of vermis	5.4429	< 0.0001	6	-64	-10
Right medial orbitral gyrus	5.4422	< 0.0001	24	34	-18
Left inferior temporal gyrus	5.4407	< 0.0001	-50	-16	-34
Left precentral gyrus	5.4392	< 0.0001	-42	8	34
Right amygdala	5.3713	< 0.0001	18	2	-16

			Right anterior orbitral gyrus	5.3632	< 0.0001	26	34	-18
			Right postcentral gyrus	5.3362	< 0.0001	64	-14	18
			Left crus II	5.3252	< 0.0001	-4	-74	-38
			Left fusiform gyrus	5.3011	< 0.0001	-24	-60	-14
			Right lobule VIII	5.2776	< 0.0001	6	-64	-44
			Left posterior orbitral gyrus	5.2722	< 0.0001	-28	12	-22
			Right precental gyrus	5.2714	< 0.0001	54	-4	44
			Right superior temporal pole	5.2402	< 0.0001	44	2	-12
			Right middle temporal gyrus	5.2269	< 0.0001	54	-16	-10
			Left supramarginal gyrus	5.2102	< 0.0001	-54	-22	14
			Right supramarginal gyrus	5.2015	< 0.0001	62	-26	26
			Right cuneus	5.1818	< 0.0001	8	-68	26
			Left crus I	5.1787	< 0.0001	-40	-50	-42
			Left amygdala	5.1542	< 0.0001	-24	4	-18
			Left postcentral gyrus	5.0990	< 0.0001	-54	-20	14
			Left cuneus	5.0843	< 0.0001	-4	-66	22
			Lobule VIII of vermis	5.0608	< 0.0001	6	-64	-42
			Right lenticular nucleus, putamen	4.8849	< 0.0001	34	6	4
			Right middle occipital gyrus	4.7680	< 0.0001	46	-72	24
			Left superior frontal gyrus, dorsolateral	4.6682	< 0.0001	-18	64	-4
			Right angular gyrus	4.6567	< 0.0001	46	-70	30
			Right middle frontal gyrus	4.6517	< 0.0001	44	30	34
			Left lobule X	4.5977	< 0.0001	-14	-36	-46
			Left supplementary motor area	4.4802	< 0.0001	-6	-10	48
			Right inferior occipital gyrus	4.4340	< 0.0001	42	-78	-10
			Lobule VII of vermis	4.4310	< 0.0001	-2	-74	-32
			Left middle occipital gyrus	4.3756	< 0.0001	-34	-88	12
			Right lateral orbitral gyrus	4.2375	< 0.0001	48	26	-14
			Right crus II	4.2156	< 0.0001	38	-44	-40
			Left anterior orbitral gyrus	4.2035	< 0.0001	-30	38	-14
			Right lobule VIIB	4.1689	< 0.0001	42	-50	-44
			Right supplementary motor area	4.1658	< 0.0001	6	10	46
			Right superior frontal gyrus, dorsolateral	4.1322	< 0.0001	26	66	10
			Right superior occipital gyrus	4.1068	< 0.0001	26	-90	14
			Right middle temporal pole	4.0441	0.0001	32	18	-34
			Left inferior parietal gyrus	3.8874	0.0001	-56	-24	38
			Left lateral orbitral gyrus	3.5784	0.0003	-36	44	-16

(D) PD vs. MSA

Contrast	Cluster	Cluster size	Area	*t* value	*p* value	Coordinates (mm)		
						x	*y*	*z*
	1	18080	Right lobule VI	9.1107	< 0.0001	30	-62	-24
			Lobule IX of vermis	8.1123	< 0.0001	0	-58	-44
			Left lobule VIII	8.0500	< 0.0001	-20	-64	-56
			Left lobule IX	8.0270	< 0.0001	2	-58	-44
			Right lobule IX	8.0100	< 0.0001	-2	-58	-46
			Right crus I	8.0086	< 0.0001	38	-48	-32
			Left lobule VI	7.9754	< 0.0001	-30	-62	-24
			Right lobule VIII	7.7580	< 0.0001	40	-56	-50
			Right lobule VIIB	7.7332	< 0.0001	42	-56	-48
PD > MSA			Right crus II	7.6078	< 0.0001	42	-58	-46
			Left lobule IV, V	7.5052	< 0.0001	12	-52	-12
			Lobule IV, V of vermis	6.9782	< 0.0001	6	-54	-8
			Left lobule VIIB	6.9535	< 0.0001	-22	-74	-52
			Right lobule IV, V	6.9032	< 0.0001	-24	-48	-20
			Left crus II	6.8097	< 0.0001	-42	-62	-46
			Lobule VIII of vermis	6.7673	< 0.0001	4	-64	-44
			Left crus I	6.6238	< 0.0001	-36	-60	-28
			Left fusiform gyrus	6.6006	< 0.0001	-26	-46	-20
			Right fusiform gyrus	6.4264	< 0.0001	28	-58	-18

			Lobule VI of vermis	6.2507	< 0.0001	0	-64	-10
			Lobule VII of vermis	5.8083	< 0.0001	2	-74	-22
			Left lingual gyrus	5.7895	< 0.0001	-12	-48	-10
			Right lingual gyrus	5.7442	< 0.0001	16	-58	-12
			Right lobule III	5.6535	< 0.0001	8	-44	-14
			Lobule III of vermis	5.6443	< 0.0001	6	-46	-14
			Left lobule III	5.1798	< 0.0001	-8	-38	-22
			Right lobule X	4.9868	< 0.0001	20	-38	-48
			Lobule X of vermis	4.8957	< 0.0001	-2	-44	-40
			Lobule I, II of vermis	4.6533	< 0.0001	-4	-40	-22
PD < MSA	1	288	Right lobule IV, V	6.6549	< 0.0001	-18	-46	-28
			Left lobule VIII	5.0064	< 0.0001	-20	-56	-44
			Left lobule VI	4.9080	< 0.0001	-22	-52	-30
			Right lobule IX	4.8443	< 0.0001	-18	-54	-44
	2	285	Right lobule VI	5.4673	< 0.0001	24	-52	-32
			Left lobule IV, V	5.0053	< 0.0001	24	-44	-30
			Right lobule VIII	4.5025	< 0.0001	10	-66	-30

(E) PD vs. PSP

Contrast	Cluster	Cluster size	Area	*t* value	*p* value	Coordinates (mm)		
						x	*y*	*z*
PD > PSP	1	11980	Left thalamus	7.0430	< 0.0001	0	-12	8
			Right thalamus	6.8338	< 0.0001	2	-14	8
			Right crus II	6.6546	< 0.0001	48	-54	-44
			Right lobule VIII	6.1329	< 0.0001	40	-46	-50
			Left lobule VIII	6.1064	< 0.0001	-40	-52	-54
			Right lobule VI	5.9920	< 0.0001	40	-44	-32
			Left lobule VI	5.9832	< 0.0001	-8	-68	-16
			Right crus I	5.9750	< 0.0001	40	-46	-32
			Right lobule VIIB	5.9378	< 0.0001	42	-50	-46
			Left crus II	5.9081	< 0.0001	-46	-58	-44
			Left lobule III	5.8639	< 0.0001	-4	-38	-10
			Left lobule VIIB	5.8581	< 0.0001	-44	-56	-56
			Lobule III of vermis	5.7555	< 0.0001	-2	-38	-12
			Left crus I	5.5612	< 0.0001	-38	-50	-30
			Lobule VI of vermis	5.2396	< 0.0001	-2	-70	-14
			Right lobule IV, V	5.0947	< 0.0001	-32	-32	-30
			Left lingual gyrus	5.0175	< 0.0001	-8	-34	-2
			Left lobule IV, V	4.9482	< 0.0001	10	-56	-10
			Lobule IV, V of vermis	4.9076	< 0.0001	6	-58	-8
			Right caudate nucleus	4.7784	< 0.0001	14	0	18
			Right lobule IX	4.7375	< 0.0001	-4	-56	-50
			Left fusiform gyrus	4.6305	< 0.0001	-30	-32	-28
			Left hippocampus	4.4377	< 0.0001	-14	-30	-8
			Right lobule X	4.3976	< 0.0001	28	-36	-46
			Right lingual gyrus	4.3080	< 0.0001	14	-62	-12
			Left lobule IX	4.1207	< 0.0001	2	-58	-48
			Right olfactory cortex	4.1177	< 0.0001	4	14	-16
			Right fusiform gyrus	4.0671	< 0.0001	24	-44	-18
			Lobule VIII of vermis	4.0610	< 0.0001	0	-74	-40
			Lobule VII of vermis	3.9979	0.0001	-2	-76	-20
			Left parahippocampal gyrus	3.9792	0.0001	-14	-30	-10
			Left caudate nucleus	3.9676	0.0001	-10	10	8
			Lobule IX of vermis	3.9576	0.0001	0	-58	-44
			Right lobule III	3.9001	0.0001	8	-36	-14
			Right gyrus rectus	3.8974	0.0001	2	18	-18
			Left superior temporal pole	3.8174	0.0001	-24	8	-30
			Left olfactory cortex	3.7358	0.0001	0	16	-10
	2	533	Left inferior frontal gyrus, triangular	4.6071	< 0.0001	-52	28	26

			Left middle frontal gyrus	4.4215	< 0.0001	-50	40	18
			Left insula	4.0807	< 0.0001	-36	14	2
			Left inferior frontal gyrus, opercular	3.6222	0.0002	-56	18	14
	3	530	Right middle frontal gyrus	4.7465	< 0.0001	48	30	36
			Right inferior frontal gyrus, triangular	4.3963	< 0.0001	56	34	18
			Right inferior frontal gyrus, orbital	3.5523	0.0003	54	42	-4
	4	378	Right precental gyrus	4.5385	< 0.0001	58	-2	46
			Right middle frontal gyrus	4.2820	< 0.0001	46	12	40
			Right inferior frontal gyrus, opercular	4.2131	< 0.0001	46	12	38
			Right postcentral gyrus	3.7511	0.0001	62	0	38
	5	304	Left middle cingulate gyrus	4.3084	< 0.0001	0	-10	40
			Right middle cingulate gyrus	3.9428	0.0001	2	-10	40
			Right supplementary motor area	3.9119	0.0001	2	-4	48
			Left supplementary motor area	3.4875	0.0003	0	0	48
	6	291	Right insula	5.8540	< 0.0001	40	-12	0
			Right superior temporal gyrus	4.0758	< 0.0001	42	-16	0
			Right rolandic operculum	3.9122	0.0001	42	-12	16

(F) MSA vs. PSP

**Contrast**	**Cluster**	**Cluster size**	**Area**	***t* value**	***p* value**	**Coordinates (mm)**		
						x	***y***	***z***
	1	1886	Right precental gyrus	5.2991	< 0.0001	58	-2	46
			Right postcentral gyrus	4.8217	< 0.0001	60	-14	46
			Right superior temporal pole	4.6838	< 0.0001	62	6	2
			Right middle frontal gyrus	4.6609	< 0.0001	54	36	20
			Right inferior frontal gyrus, triangular	4.6178	< 0.0001	54	34	20
			Right rolandic operculum	4.4789	< 0.0001	62	6	4
			Right anterior orbitral gyrus	4.4203	< 0.0001	36	46	-18
			Right inferior frontal gyrus, opercular	4.4002	< 0.0001	50	18	26
			Right medial orbitral gyrus	4.0883	0.0001	22	40	-24
			Right inferior frontal gyrus, orbital	3.7841	0.0001	46	48	-12
			Right lateral orbitral gyrus	3.6960	0.0002	48	46	-14
	2	1076	Left anterior orbitral gyrus	5.5983	< 0.0001	-20	56	-18
			Left medial orbitral gyrus	5.4785	< 0.0001	-12	62	-18
			Left gyrus rectus	4.5945	< 0.0001	-6	44	-26
			Right gyrus rectus	4.4537	< 0.0001	4	36	-18
			Left middle frontal gyrus	4.4264	< 0.0001	-24	60	-12
			Left superior frontal gyrus, medial orbital	3.9584	0.0001	-8	64	-14
			Left superior frontal gyrus, dorsolateral	3.8887	0.0001	-24	62	-10
	3	948	Left precental gyrus	5.7577	< 0.0001	-60	8	24
MSA > PSP			Left inferior frontal gyrus, opercular	5.5051	< 0.0001	-62	10	24
			Left inferior frontal gyrus, triangular	4.9697	< 0.0001	-52	38	14
			Left middle frontal gyrus	4.1718	< 0.0001	-50	40	16
			Left rolandic operculum	4.1141	< 0.0001	-62	4	6
			Left insula	3.5809	0.0003	-42	18	4
	4	599	Right thalamus	4.8039	< 0.0001	4	-20	10
			Left thalamus	4.6405	< 0.0001	0	-14	8
	5	375	Right inferior temporal gyrus	4.5699	< 0.0001	48	-74	-10
			Right inferior occipital gyrus	4.5279	< 0.0001	48	-76	-10
			Right middle temporal gyrus	4.0612	0.0001	64	-54	-8
			Right fusiform gyrus	3.9838	0.0001	52	-68	-18
			Right lingual gyrus	3.7083	0.0002	38	-88	-18
	6	299	Left postcentral gyrus	5.2319	< 0.0001	-64	-10	26
			Left supramarginal gyrus	4.6745	< 0.0001	-64	-22	26
	7	296	Left superior frontal gyrus, medial	4.1756	< 0.0001	-2	38	34
			Left anterior cingulate gyrus	3.9404	0.0001	-8	50	10
			Right anterior cingulate gyrus	3.9300	0.0001	6	50	8
			Right superior frontal gyrus, medial	3.8749	0.0001	6	52	6
			Right superior frontal gyrus, medial orbital	3.7071	0.0002	8	54	-4

	8	291	Left middle cingulate gyrus	4.2905	< 0.0001	0	-42	34
			Right middle cingulate gyrus	4.2293	< 0.0001	2	-42	34
			Left posterior cingulate gyrus	4.0571	0.0001	0	-42	32
MSA < PSP	1	355	Left lobule IX	4.2718	< 0.0001	8	-54	-46
			Right lobule VIIB	4.1113	< 0.0001	20	-74	-44
			Right lobule VIII	4.0808	0.0001	20	-72	-46
			Right crus II	3.6445	0.0002	22	-76	-44

Table S3. Areas of differences in regional homogeneity between groups, corresponding to Figures S3 and S4.

(A) Healthy controls (HC) vs. multiple system atrophy (MSA)

**Contrast**	**Cluster**	**Cluster size**	**Area**	***t* value**	***p* value**	**Coordinates (mm)**		
						x	***y***	***z***
HC > MSA	1	328	Left crus I	4.0209	0.0001	-32	-60	-32
			Left crus II	4.0074	0.0001	-18	-78	-34
			Left lobule VI	3.9856	0.0001	-32	-58	-32

Table S4. Areas of differences in degree centrality between groups, corresponding to Figures S5 and S6.

(A) Healthy controls (HC) vs. Parkinson's disease (PD)

Contrast	Cluster	Cluster size	Area	*t* value	*p* value	Coordinates (mm)		
						x	*y*	*z*
HC > PD	1	1953	Right superior temporal gyrus	5.4734	< 0.0001	62	-18	10
			Right rolandic operculum	5.3382	< 0.0001	62	-18	12
			Right postcentral gyrus	4.6055	< 0.0001	56	-12	20
			Right supramarginal gyrus	4.4227	< 0.0001	54	-14	24
			Right precental gyrus	4.0455	< 0.0001	54	-14	42
	2	954	Left precental gyrus	5.3383	< 0.0001	-50	-2	20
			Left postcentral gyrus	5.1405	< 0.0001	-50	-4	18
			Left rolandic operculum	4.6906	< 0.0001	-50	-2	16
			Left superior temporal gyrus	4.6242	< 0.0001	-56	-22	6
			Left transverse temporal gyrus	4.1975	< 0.0001	-54	-16	8
	3	546	Left middle cingulate gyrus	4.5426	< 0.0001	-10	-8	40
			Right middle cingulate gyrus	4.5173	< 0.0001	10	-4	42
			Left supplementary motor area	4.0448	< 0.0001	-10	-2	46
			Right supplementary motor area	3.9651	0.0001	10	-4	46
	4	393	Left postcentral gyrus	4.0561	< 0.0001	-42	-12	44
			Left precental gyrus	4.0249	< 0.0001	-40	-10	44
			Left inferior parietal gyrus	3.6751	0.0002	-44	-26	44

(B) HC vs. progressive supranuclear palsy (PSP)

Contrast	Cluster	Cluster size	Area	*t* value	*p* value	Coordinates (mm)		
						x	*y*	*z*
HC > PSP	1	770	Left middle cingulate gyrus	5.5133	< 0.0001	-4	-8	42
			Left supplementary motor area	4.5556	< 0.0001	-2	-12	48
			Right middle cingulate gyrus	4.5481	< 0.0001	2	-14	44
			Left anterior cingulate gyrus	4.0690	0.0001	-2	16	28
			Right anterior cingulate gyrus	3.8716	0.0001	2	16	28

Table S5. Counts of grey matter (GM) regions that contributed to classification problems. Involved GM regions were segregated into cerebral (Cbrm) and cerebellar (Cbll) regions, and the respective GM regions were counted for each of GM volume, regional homogeneity (ReHo), and degree centrality (DegCen). The 'Total' column represents counts of GM regions involved in any of the three measures, with values within parentheses corresponding to counts of GM regions involved in both the structural measure (GM volume) and more than one of the functional measures (ReHo and DegCen).

	Total		GM volume		ReHo		DegCen	
	Cbrm	Cbll	Cbrm	Cbll	Cbrm	Cbll	Cbrm	Cbll
HC vs. PD	60 (14)	9 (3)	45	5	8	4	22	5
HC vs. MSA	24 (1)	25 (14)	3	25	12	8	11	10
HC vs. PSP	91 (28)	26 (9)	91	26	19	8	11	2
PD vs. MSA	30 (2)	24 (17)	15	24	9	4	8	17
PD vs. PSP	70 (4)	25 (5)	61	25	10	0	3	5
MSA vs. PSP	87 (28)	12 (0)	84	4	23	2	8	8

HC, healthy controls; PD, Parkinson's disease; MSA, multiple system atrophy; and PSP, progressive supranuclear palsy.

Figure S1. Voxel-wise statistical comparisons of gray matter (GM) volume between groups. For voxels of significant group differences, *t* values computed from two sample *t*-tests were mapped to colors. Positive and negative *t* values mean being higher and lower, respectively, of GM volume in the latter group than in the former group. HC, healthy controls; PD, Parkinson's disease; MSA, multiple system atrophy; and PSP, progressive supranuclear palsy.

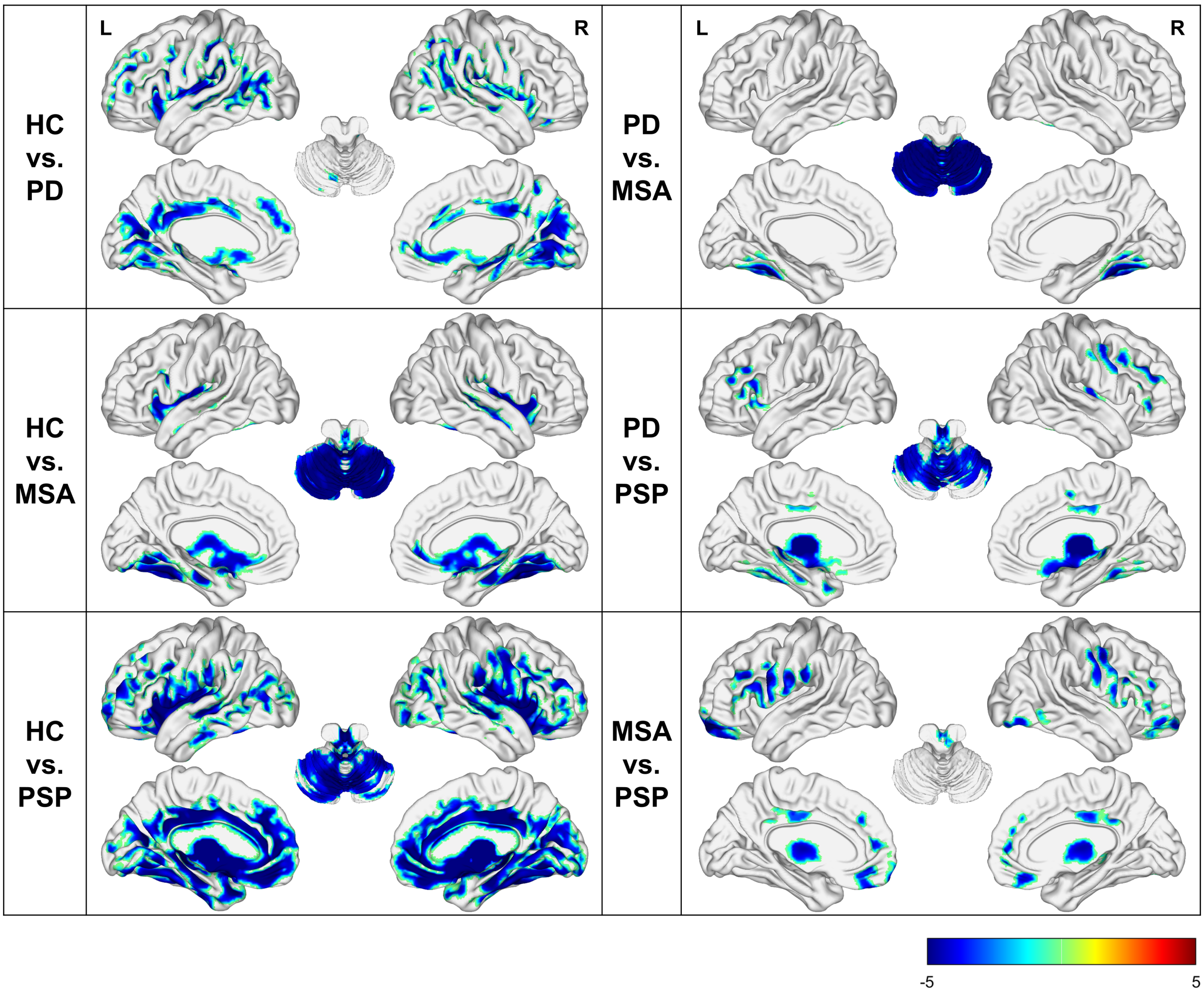

Figure S2. Axial slice views of Figure S1. Slices are oriented according to the neurological convention. HC, healthy controls; PD, Parkinson's disease; MSA, multiple system atrophy; and PSP, progressive supranuclear palsy.

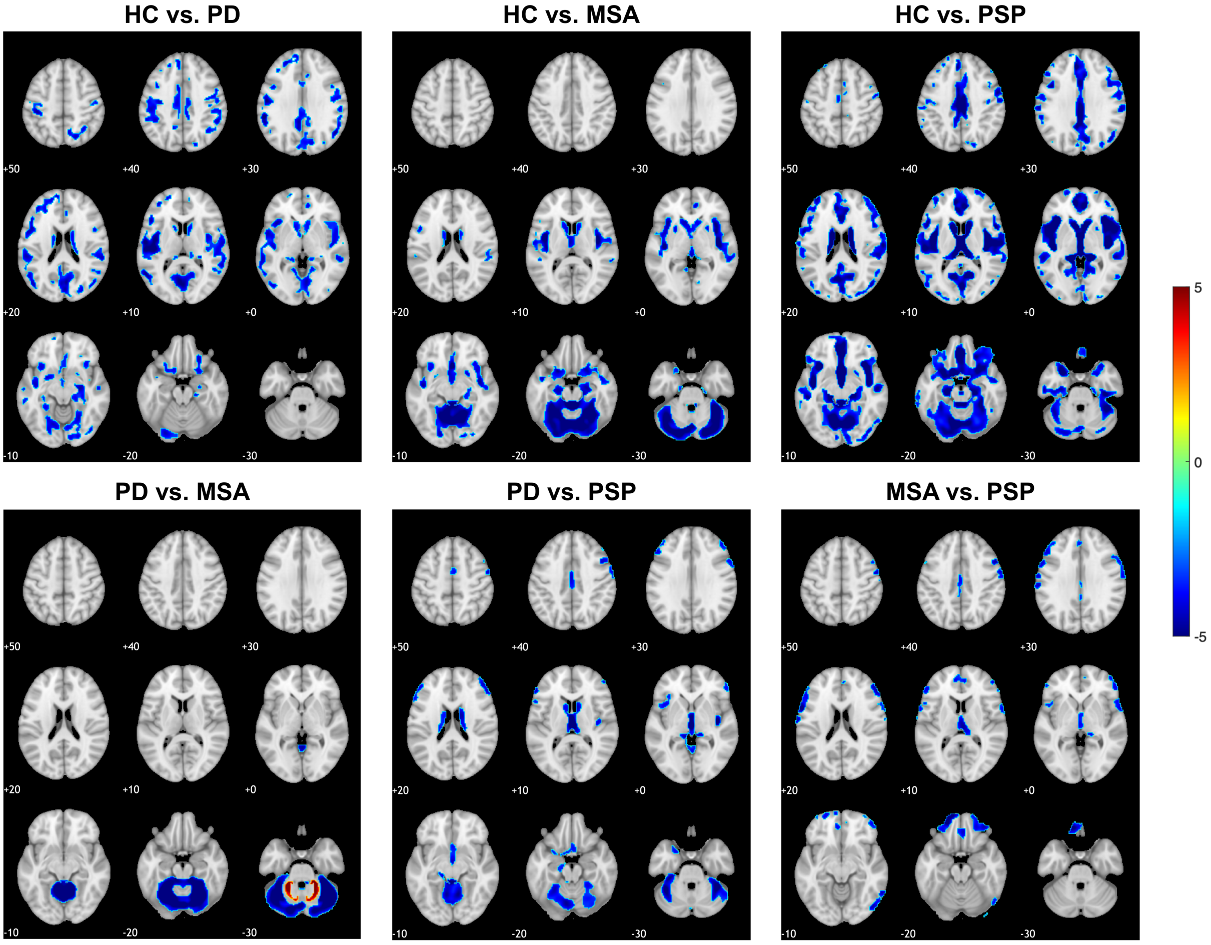

Figure S3. Voxel-wise statistical comparisons of regional homogeneity (ReHo) between groups. For voxels of significant group differences, *t* values computed from two sample *t*-tests were mapped to colors. Positive and negative *t* values mean being higher and lower, respectively, of ReHo in the latter group than in the former group. Group comparisons were left blank if there were no statistically significant differences for those. HC, healthy controls; PD, Parkinson's disease; MSA, multiple system atrophy; and PSP, progressive supranuclear palsy.

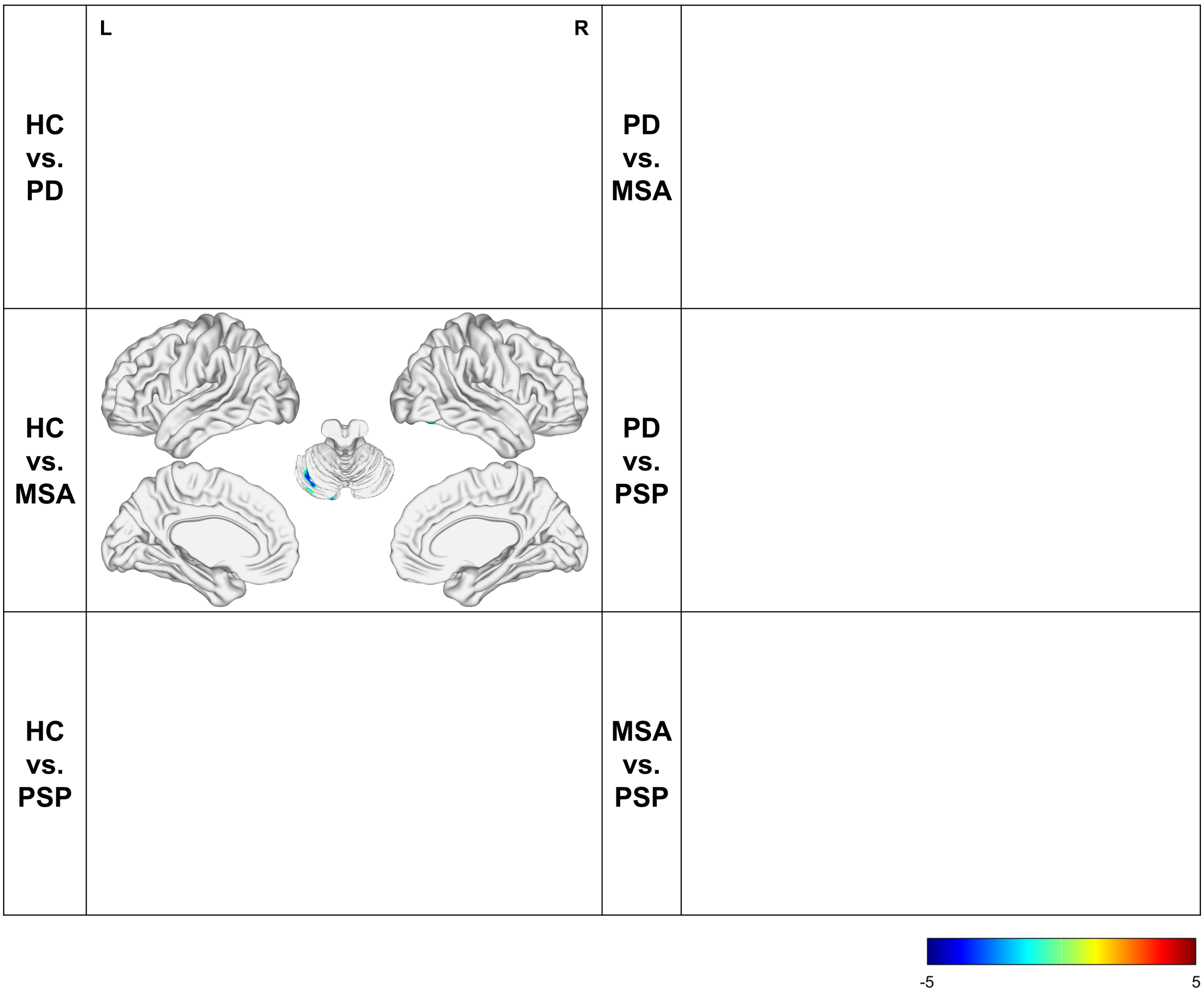

Figure S4. Axial slice views of Figure S3. Slices are oriented according to the neurological convention. HC, healthy controls; and MSA, multiple system atrophy.

HC vs. MSA

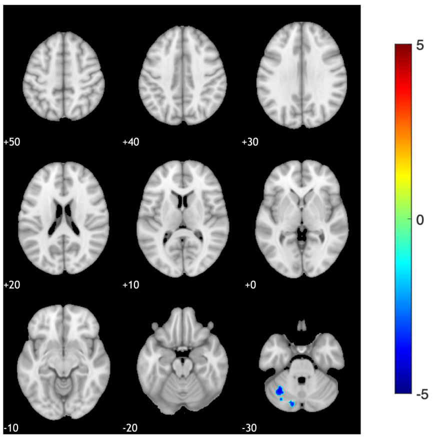

Figure S5. Voxel-wise statistical comparisons of degree centrality (DegCen) between groups. For voxels of significant group differences, t values computed from two sample t-tests were mapped to colors. Positive and negative t values mean being higher and lower, respectively, of DegCen in the latter group than in the former group. Group comparisons were left blank if there were no statistically significant differences for those. HC, healthy controls; PD, Parkinson's disease; MSA, multiple system atrophy; and PSP, progressive supranuclear palsy.

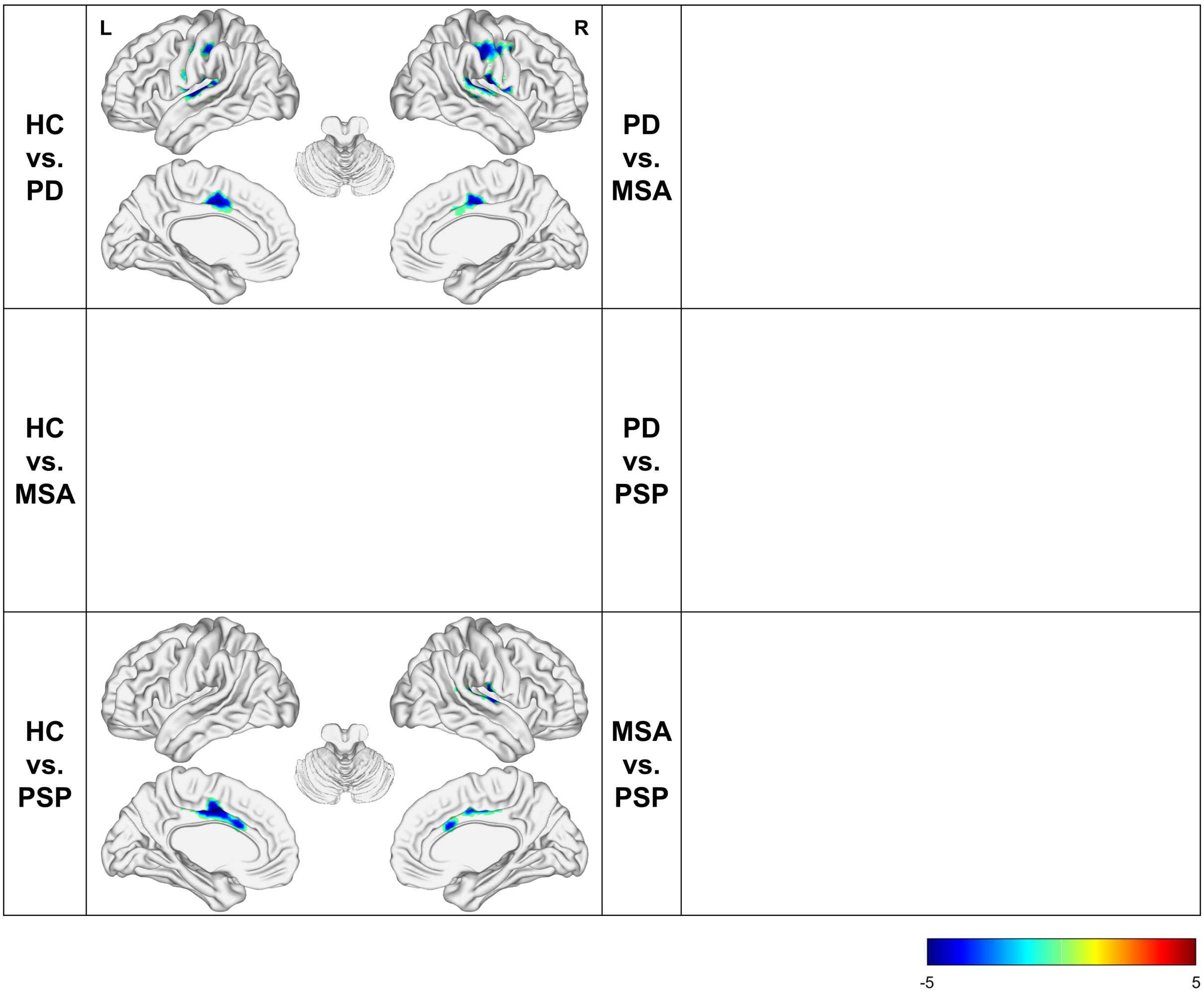

Figure S6. Axial slice views of Figure S5. Slices are oriented according to the neurological convention. HC, healthy controls; PD, Parkinson's disease; and PSP, progressive supranuclear palsy.

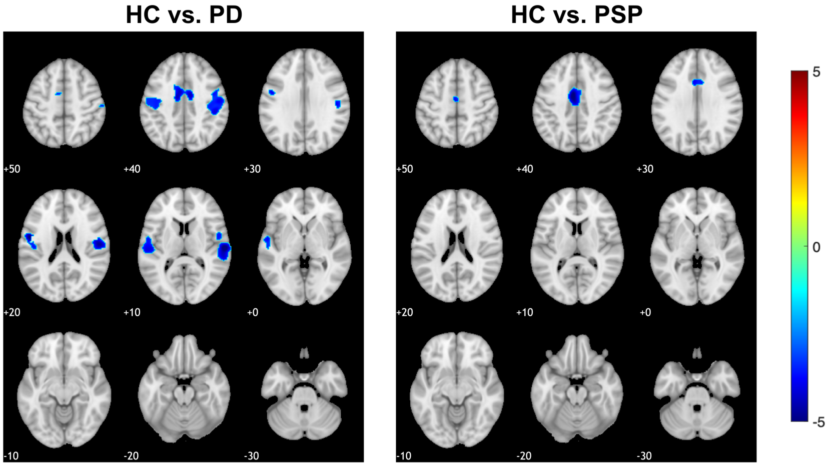

Figure S7. A heat map of the classification accuracy of support vector machine (SVM) classifiers for different classification problems. In the SVM classifiers, individual measures or combinations of those were employed as predictor sets. The considered measures included grey matter (GM) volume (Vol), regional homogeneity (ReHo), and degree centrality (DegCen), and predictor values were collected from 116 GM regions based on the automated anatomical labeling atlas (Tzourio-Mazoyer et al., 2002) for the three measures. HC, healthy controls; PD, Parkinson's disease; MSA, multiple system atrophy; and PSP, progressive supranuclear palsy.

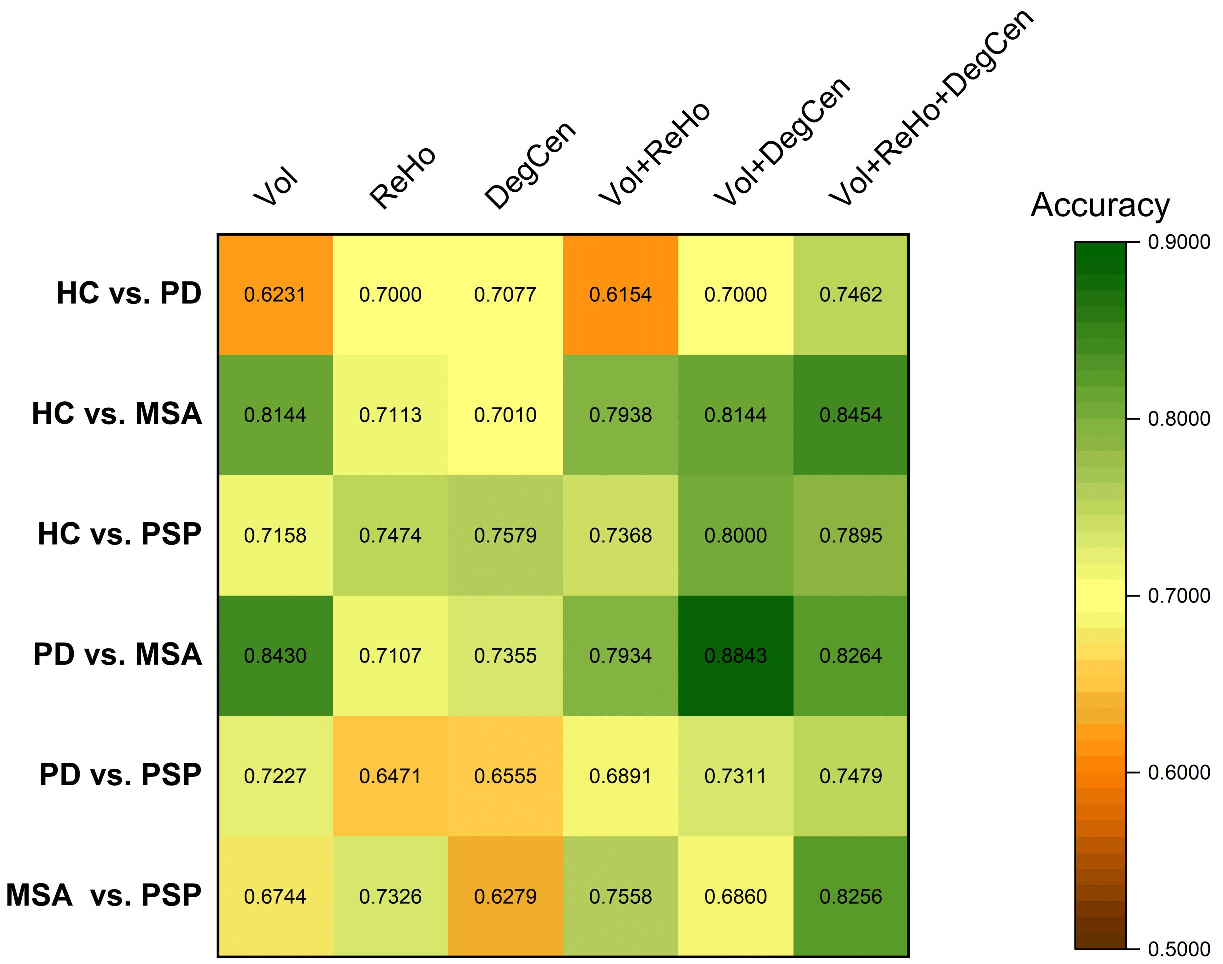

Figure S8. A heat map of the classification accuracy of support vector machine (SVM) classifiers for different classification problems. In the SVM classifiers, individual measures or combinations of those were employed as predictor sets. The considered measures included grey matter (GM) volume (Vol), regional homogeneity (ReHo), and degree centrality (DegCen), and predictor values were collected from 60 GM regions based on the Hammers atlas (Hammers et al., 2003) for the three measures. HC, healthy controls; PD, Parkinson's disease; MSA, multiple system atrophy; and PSP, progressive supranuclear palsy.

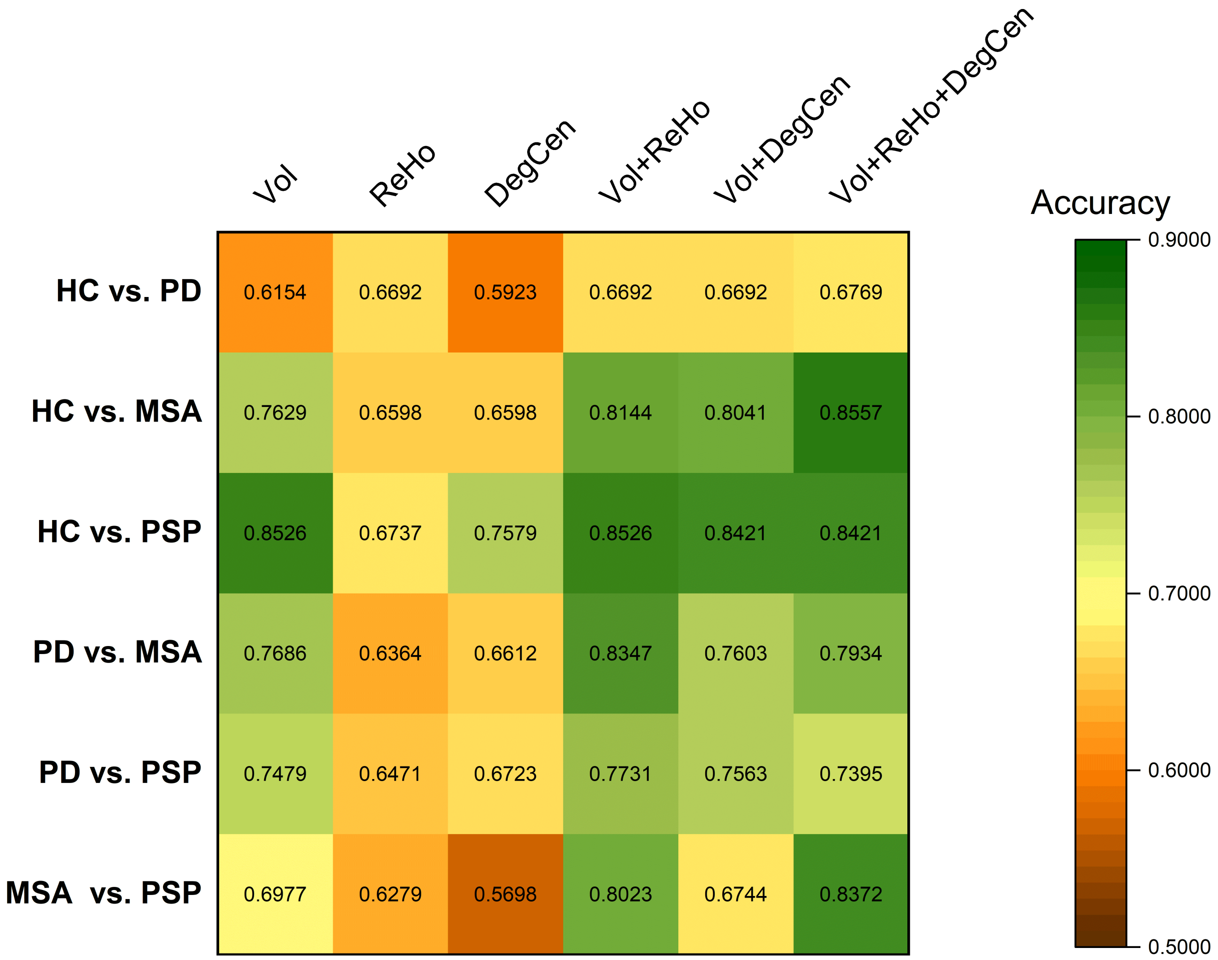

References

Hammers, A., Allom, R., Koepp, M. J., Free, S. L., Myers, R., Lemieux, L., . . . Duncan, J. S. (2003). Three-dimensional maximum probability atlas of the human brain, with particular reference to the temporal lobe. *Human Brain Mapping, 19*(4), 224-247. doi: 10.1002/hbm.10123

Lee, H. W., Arora, J., Papademetris, X., Tokoglu, F., Negishi, M., Scheinost, D., . . . Constable, R. T. (2014). Altered functional connectivity in seizure onset zones revealed by fMRI intrinsic connectivity. *Neurology, 83*(24), 2269-2277. doi:10.1212/wnl.0000000000001068

Rolls, E. T., Joliot, M., & Tzourio-Mazoyer, N. (2015). Implementation of a new parcellation of the orbitofrontal cortex in the automated anatomical labeling atlas. *NeuroImage, 122*, 1-5. doi:10.1016/j.neuroimage.2015.07.075

Tzourio-Mazoyer, N., Landeau, B., Papathanassiou, D., Crivello, F., Etard, O., Delcroix, N., . . . Joliot, M. (2002). Automated Anatomical Labeling of Activations in SPM Using a Macroscopic Anatomical Parcellation of the MNI MRI Single-Subject Brain. *NeuroImage, 15*(1), 273-289. doi:10.1006/nimg.2001.0978

Zang, Y., Jiang, T., Lu, Y., He, Y., & Tian, L. (2004). Regional homogeneity approach to fMRI data analysis. *NeuroImage, 22*(1), 394-400. doi:10.1016/j.neuroimage.2003.12.030
